# Knowledge-Guided Explainable Recommendation Tool for Cancer Risk Prediction Models Using Retrieval-Augmented Large Language Models: Development and Validation Study

**DOI:** 10.2196/78519

**Published:** 2026-03-09

**Authors:** Shumin Ren, Xin Zheng, Jing Zhao, Jiale Du, Yuxin Zhang, Cheng Bi, Jie Song, Jinyi Zhang, Hongmei Lang, Fan Zhang, Bairong Shen

**Affiliations:** 1 Institutes for Systems Genetics, West China Hospital of Sichuan University, Frontiers Science Center for Disease-related Molecular Network, Chengdu, Sichuan, 610041, China, 86 15995854635; 2 West China School of Medicine, Sichuan University, Sichuan University affiliated Chengdu Second People's Hospital, Chengdu Second People's Hospital, Chengdu, Sichuan, China; 3 Health Management Center, General Practice Medical Center, West China Hospital, Sichuan University, Chengdu, Sichuan, China

**Keywords:** cancer risk prediction models, large language models, LLMs, retrieval-augmented generation, RAG, personalized medicine, biomedical information retrieval

## Abstract

**Background:**

Cancer risk prediction models are vital for precision prevention, enabling individualized assessment of cancer susceptibility based on genetic, clinical, environmental, and lifestyle factors. However, the practical use of these models is hindered by fragmented resources, heterogeneous reporting, and the absence of transparent, structured systems for systematic discovery and comparison.

**Objective:**

This study aimed to develop a retrieval-augmented, knowledge-guided system that provides accurate recommendations for cancer risk prediction models.

**Methods:**

We developed CanRisk-RAG, a recommendation platform underpinned by a precisely constructed knowledge base comprising more than 800 peer-reviewed cancer risk prediction models spanning diverse cancer types, modeling approaches, and predictive variables. The system integrates (1) large language model (LLM)–based semantic tag extraction, (2) embedding vectorization of structured metadata and abstracts, (3) a multifactor ranking algorithm combining semantic similarity with multiple quality indicators, and (4) LLM-generated literature summarization to support rapid user interpretation. Performance was evaluated across 4 types of representative queries. Eight domain experts independently assessed retrieval quality. CanRisk-RAG was benchmarked against PubMed, ChatGPT-4o, ScholarAI, and Gemini 1.5 Flash.

**Results:**

On the independent validation set, CanRisk-RAG consistently outperformed all 4 baseline applications, achieving the highest overall relevance (8.30 [SD 0.59]) and reliability (7.62 [SD 0.76]) scores on a 10-point scale (*P*<.05). It also demonstrated high authenticity, data completeness, and consistency. Baseline applications frequently returned incomplete, inconsistent, or fabricated results, especially for complex, multifactorial queries, whereas CanRisk-RAG delivered accurate and structured recommendations grounded in validated evidence.

**Conclusions:**

CanRisk-RAG presents a transparent, domain-specific, and semantically enriched framework for discovering cancer risk prediction models, addressing several limitations of existing keyword-based search tools and general-purpose LLMs. By integrating structured knowledge, multifactor ranking, and LLM-based reasoning, the system aims to improve the precision, reproducibility, and usability of model selection in cancer risk prediction. While our evaluation demonstrates encouraging performance compared with baseline systems, further validation in broader clinical contexts and real-world applications is warranted. The framework’s general design may also be adaptable to other clinical model domains, providing a potential foundation for advancing evidence-based model discovery in precision medicine.

## Introduction

Cancer risk prediction models are increasingly recognized as essential components of precision medicine, offering clinicians and researchers the ability to estimate an individual’s likelihood of developing cancer based on diverse risk factors. These models integrate genetic markers, clinical data, environmental exposures, and lifestyle behaviors, thereby enabling more personalized approaches to prevention and screening [1-4]. These heterogeneous factors are not independent but often interact in complex and nonlinear ways. For example, genetic susceptibility may amplify the carcinogenic effects of smoking or obesity, while lifestyle interventions can partially mitigate inherited risk [5,6]. Understanding and modeling these interdependencies is therefore crucial for accurate cancer risk prediction.

Statistical approaches such as logistic regression, Cox proportional hazards models, and Bayesian networks have traditionally been used to analyze correlations and interactions among these predictors. More recently, advanced machine learning techniques, including random forests, gradient boosting, and deep neural networks, have enabled the modeling of higher-order, nonlinear, and cross-domain relationships between genetic, clinical, and environmental factors [7-10]. These methods can capture subtle patterns that traditional regression frameworks may overlook, thus improving predictive accuracy and interpretability when properly validated.

By identifying individuals at elevated risk, cancer risk prediction models facilitate earlier interventions, targeted monitoring, and ultimately reductions in cancer incidence and mortality [11,12]. Recent advances have demonstrated that combining heterogeneous risk determinants can substantially improve predictive accuracy. For instance, multivariate models that combine germline mutations (eg, BRCA1/2), hormonal and reproductive history, and lifestyle exposures have achieved strong discriminative power in breast cancer risk prediction [13,14], while integrative models linking smoking intensity, air pollution exposure, and genomic markers have improved lung cancer risk stratification [15,16]. Together, these developments highlight both the promise and the complexity of synthesizing genetic, clinical, environmental, and behavioral information into actionable tools for precision prevention.

Despite the promise of cancer risk prediction models, their practical impact is limited by barriers related to accessibility and adoption. A fundamental problem lies in the fragmentation of available models, which vary widely in methodological approach, target population, and predictive variables. Clinicians and researchers often struggle to identify the most appropriate model for a given use case, a challenge compounded by the lack of centralized, structured resources for systematic discovery. General medical retrieval systems are ill-suited to this task, as they are designed primarily to locate literature rather than extract or recommend specialized prediction tools. As a result, existing models are underutilized, research efforts are frequently duplicated, and the translational pathway from model development to clinical implementation remains inefficient. Indeed, it has been observed that most published prediction models are never adopted in clinical practice, highlighting a persistent gap between academic research and implementation [17]. Reviews further emphasize that although many models have been developed, only a small fraction have been successfully translated into clinical or public health practice, largely due to limitations in validation, usability, and acceptability [18,19].

To date, most users seeking cancer risk prediction models have relied on mainstream biomedical databases such as PubMed [20]. While PubMed provides broad access to scientific publications, its keyword-based search mechanisms often generate an overwhelming number of results, many of which are irrelevant for model identification. Moreover, critical details regarding model structure, predictive variables, validation cohorts, or performance metrics are typically buried in unstructured text, making systematic retrieval difficult.

In parallel, institutional platforms have been developed to provide more direct access to specific risk prediction tools. The US National Cancer Institute’s Division of Cancer Epidemiology and Genetics offers a set of statistical macros and online calculators covering selected cancer types [21], while the Memorial Sloan Kettering Cancer Center provides nomograms tailored to particular clinical scenarios [22]. Although these resources represent valuable contributions, their scope remains restricted to only several (typically fewer than a dozen) tools, employ relatively narrow modeling methodologies, and lack detailed metadata regarding model characteristics or generalizability.

Large language models (LLMs) have recently been explored as potential solutions to improve medical information access. Their ability to integrate knowledge and process complex clinical text holds significant promise for bridging gaps between scattered information and practical decision-making [23-26]. However, empirical studies highlight critical weaknesses in LLMs, including limited precision, the generation of fabricated or irrelevant outputs, and the opacity of reasoning processes [23,27,28]. Retrieval-augmented generation (RAG) has been proposed as a mechanism to mitigate these shortcomings by grounding model outputs in external knowledge sources [29,30]. Several initiatives illustrate the potential of this approach. RefAI [31], for instance, combines PubMed retrieval with a GPT-4–based summarization system, enabling more effective access to recent biomedical publications. Similarly, WeiseEule [32] integrates keyword-driven retrieval from PubMed with LLM-based responses to improve query precision. However, these systems largely depend on user-defined search terms and ad hoc knowledge construction, leading to inconsistent coverage and limited reproducibility. Moreover, they are not designed to handle structured, model-specific metadata, such as predictive variables, validation cohorts, or performance metrics, that are critical for evidence-based model selection.

To address these challenges, the present study introduces CanRisk-RAG, a knowledge-guided and LLM-enhanced recommendation system designed to systematically retrieve, rank, and summarize cancer risk prediction models. Built on a knowledge base that we systematically constructed to consolidate validated cancer risk prediction models across multiple cancer types, predictive variables, and methodological frameworks, CanRisk-RAG provides a structured foundation for model discovery and reuse. By combining domain-curated knowledge with LLM-driven semantic reasoning, the system bridges the gap between unstructured user queries and structured model metadata. Furthermore, a multi-factor ranking algorithm integrates semantic similarity with evidence-based indicators, such as publication quality, model performance, and timeliness, ensuring that recommended models are both relevant and scientifically robust. To enhance interpretability and usability, CanRisk-RAG also generates concise LLM-based summaries that highlight key model characteristics.

In essence, CanRisk-RAG aims to provide a scalable, transparent, and domain-aware retrieval framework that supports both research and clinical decision-making in cancer risk assessment. By facilitating the efficient reuse of existing models and improving accessibility for diverse user needs, including population-level screening and high-risk cohorts defined by genetic predisposition or family history, it represents a step toward operationalizing precision prevention in oncology.

This paper is organized as follows: We first introduce the data sources and system development workflow. Next, we describe the design of the evaluation tasks and the specific evaluation metrics used. We then present the performance results of CanRisk-RAG in terms of model recommendation and tool usability, along with comparative analyses against four baseline applications. Finally, we discuss the implications of the findings and explore potential directions for extending the system to other cancer research or medical domains.

## Methods

### Overview

The Methods section outlines the construction of CanRisk-RAG, covering data preparation, semantic vectorization, tag extraction via LLM, hybrid ranking with multiple quality indicators, literature summarization, and comparative evaluation. For more implementation details and technical specifications, please refer to Supplemental Information 1.1 in Multimedia Appendix 1.

### Dataset

#### Data Preparation

We constructed a knowledge base containing peer-reviewed cancer risk prediction models, consolidating clinically developed and validated cancer risk prediction models extracted from PubMed. The detailed search criteria and the inclusion and exclusion standards used for model collection are provided in Supplemental Information 1.2 and 1.3 in Multimedia Appendix 1.

**Figure 1.**
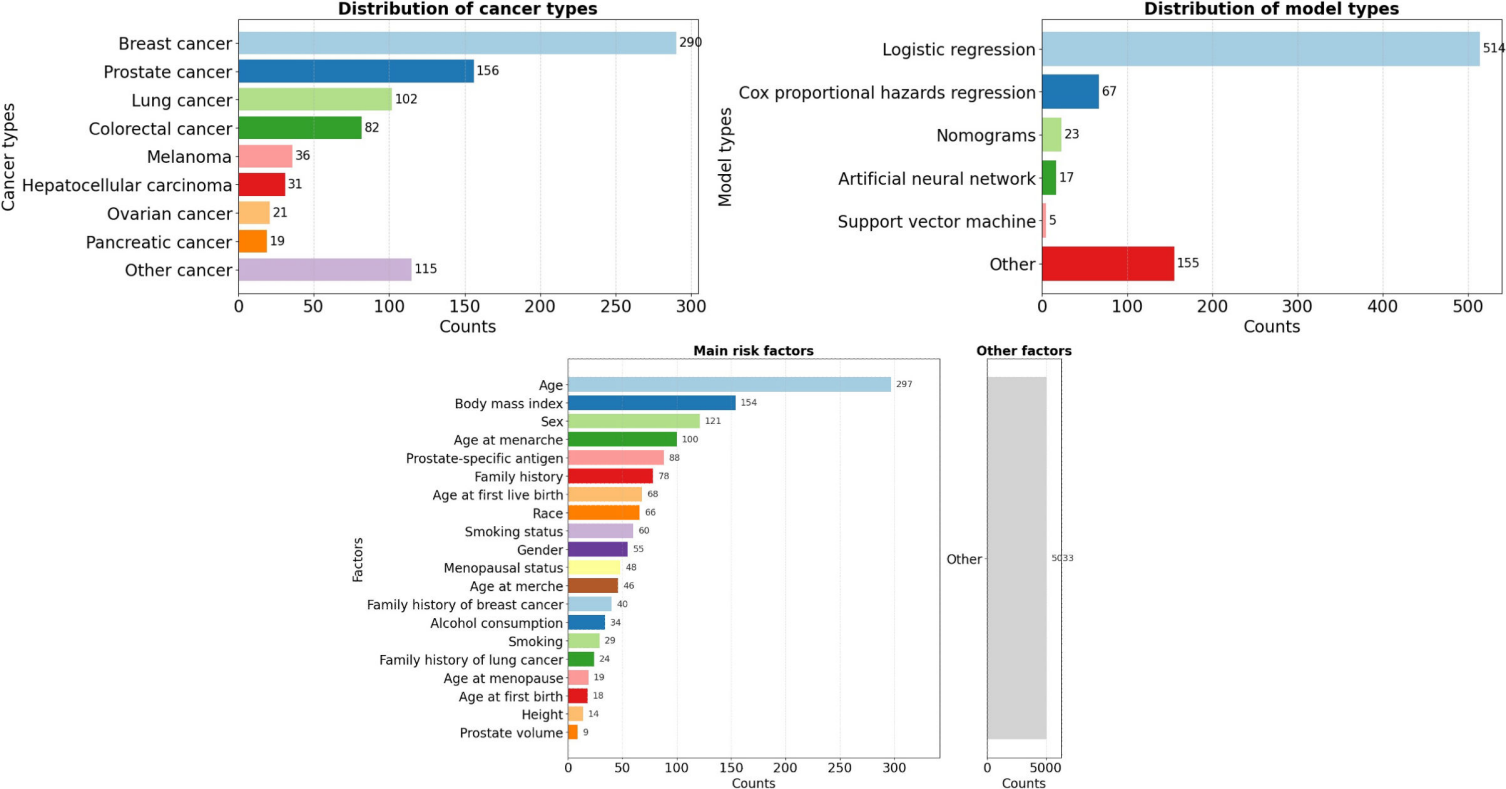
Distribution of cancer types, model types, and predictive factors in the CanRisk-RAG.

Following this procedure, a total of 808 cancer risk prediction models were included. Each model is uniquely identified by a model ID (MID). Each model was comprehensively described using metadata covering 3 major categories, namely basic information, sample information, and model information, across a total of 36 fields. To facilitate structured storage and interpretation, an Entity–Relationship (ER) diagram was constructed (see Supplemental Information 1.4 in Multimedia Appendix 1). In addition, as illustrated in Figure 1, the knowledge base organizes data across more than 10 cancer types (see Supplemental Information 2.1 in Multimedia Appendix 2 for the full distribution), over 13 methodological frameworks (eg, logistic regression, Cox regression, nomograms), and 3095 predictive factors, including age, body mass index, sex, and family history.

#### Data Segmentation and Vectorization

To enable semantic retrieval, data from the knowledge base were transformed into structured vector representations. Key attributes of each model, including cancer type, methodological approach, research stage, predictive factors, and geographic context were integrated to form composite descriptors that reflect the essential semantic characteristics of the models. These descriptors were then encoded using advanced sentence embedding techniques to generate dense vector representations, ensuring that key semantic meaning was preserved.

In parallel, the textual abstracts of the model research were independently embedded to capture complementary contextual details not fully represented by structured metadata. The combination of structured semantic vectors and unstructured textual embeddings provided a comprehensive representation of each model (see Supplemental Information 1.1 in Multimedia Appendix 1 for details). This dual encoding strategy established the basis for subsequent similarity search and model ranking, allowing the system to align user queries with the most relevant prediction models in a context-aware and interpretable manner.

### Workflow of CanRisk-RAG

#### System Architecture

**Figure 2.**
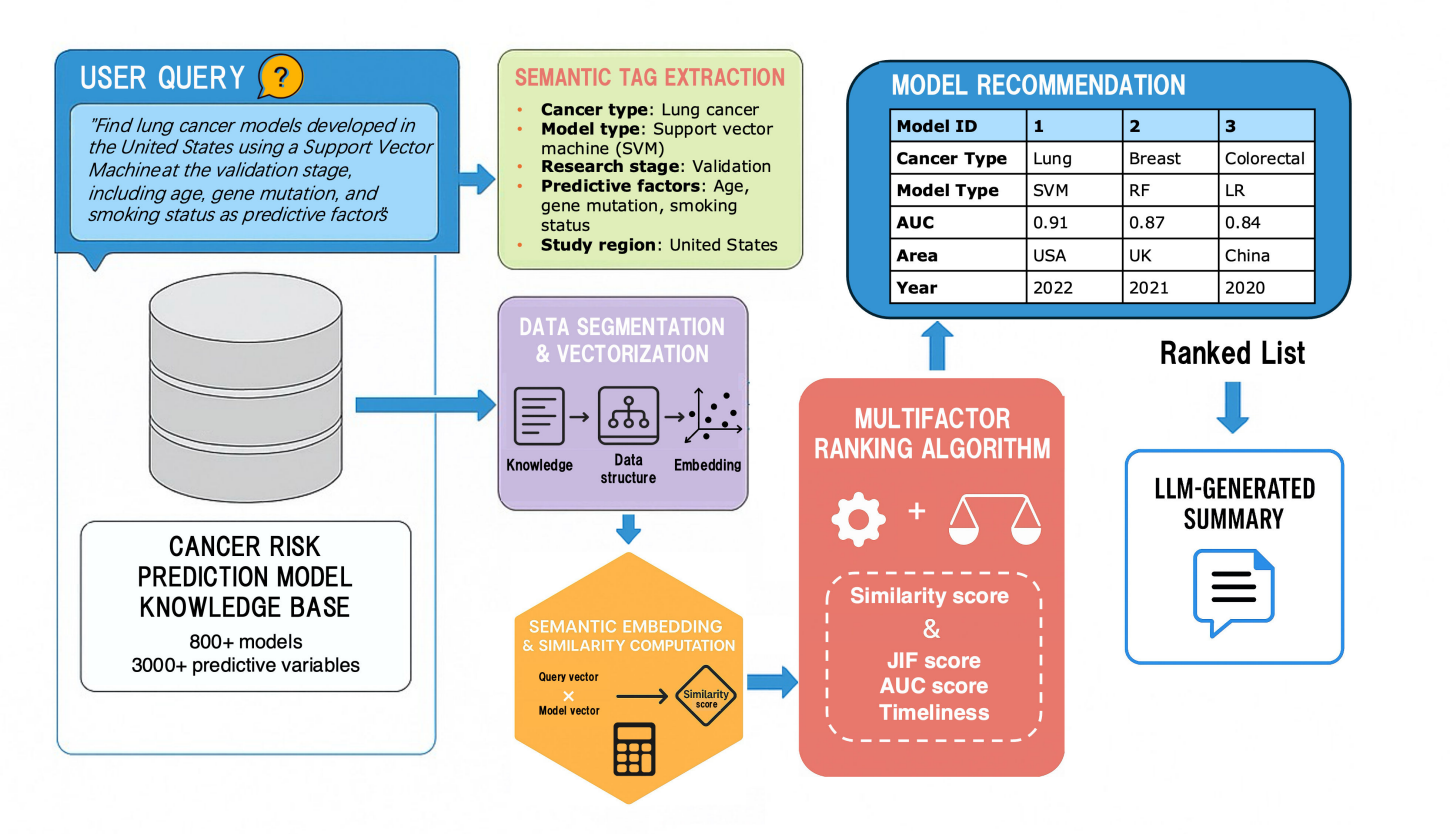
Pipeline of CanRisk-RAG construction. This figure presents the full pipeline of CanRisk-RAG, a retrieval-augmented framework designed to identify and summarize validated cancer risk prediction models from peer-reviewed resources. The workflow proceeds from user query interpretation to model recommendation: (1) users enter a natural language query describing desired model features; (2) a large language model (LLM)–based semantic tag extractor converts the query into structured fields (eg, cancer type, model type, predictive factors, study region); (3) the query is matched against a knowledge base constructed from PubMed containing over 800 cancer risk models and 3000 predictive variables; (4) structured metadata and text are vectorized using optimized sentence embeddings; (5) semantic similarity between query and models is computed; (6) a multifactor ranking algorithm integrates similarity, journal impact factor (JIF), model area under the curve (AUC), and publication recency into a composite score; and (7) top-ranked models are presented with metadata and an LLM-generated summary.

The overall workflow of CanRisk-RAG comprises a multistage pipeline that transforms unstructured model resources into structured model recommendations (Figure 2). The system integrates several key components: (1) semantic tag extraction, in which user queries are parsed by a LLM into predefined biomedical fields; (2) semantic embedding and vectorization, which encode both structured metadata and unstructured abstracts into dense representations for similarity computation; (3) multifactor ranking, combining semantic similarity with quality indicators such as the journal impact factor (JIF), the area under the curve (AUC), and publication recency to ensure reliable prioritization; and (4) LLM-based summarization, which synthesizes retrieved content into concise and readable outputs. Together, these stages enable CanRisk-RAG to perform context-aware retrieval, interpret complex user intents, and deliver transparent, evidence-based model recommendations supported by structured domain knowledge.

#### Tag Extraction for Enhanced Semantic Matching

To support efficient retrieval, user queries were first transformed from free text into structured semantic representations. This process was facilitated by a LLM (DeepSeek-V3) [33], chosen for its open-source nature and ability to support scalable deployment. Rather than directly matching raw text against the database, the model extracted predefined semantic fields, including cancer type, model type, research stage, predictive factors, and study region, thereby aligning user intent with structured knowledge (see Supplemental Information 1.5 in Multimedia Appendix 1 for the prompt contents).

An evaluation set of 40 queries (Supplemental Information 1.6 in Multimedia Appendix 1) was constructed to evaluate the tag extraction process. The queries were designed to cover diverse cancer types, model types, predictive factor combinations, geographic contexts, and noisy inputs (eg, typos and incomplete descriptions). Two independent annotators created gold labels following the predefined schema (Cancer_Type, Model_Type, Research_Type, Involved_Factors, Country), and disagreements were adjudicated by a third expert. Precision, recall, and *F*_1_-score were calculated per category and additionally provided uncertainty estimates using Wilson 95% CIs for precision (Supplemental Information 1.7 in Multimedia Appendix 1).

#### Embedding Model Selection

To prioritize relevant models, CanRisk-RAG employs a ranking strategy primarily driven by semantic similarity. User queries are semantically aligned with database entries through embedding-based similarity, which captures both structured tags and contextual meaning. The similarity score serves as the main determinant, reflecting the degree of correspondence between user intent and model characteristics.

Before performing multifactor ranking, to determine which embedding model achieved the best semantic representation performance, we evaluated four widely used sentence-embedding architectures [34-36], including all-MiniLM-L6-v2, all-MiniLM-L12-v2, all-mpnet-base-v2, and all-distilroberta-v1. Each model’s output was assessed by 2 independent evaluators across 12 predefined recommendation queries derived from 4 thematic categories (Textbox 1). For each model, average relevance and reliability scores were calculated. All-distilroberta-v1 achieved the highest overall performance, with statistically significant advantages in both relevance and reliability metrics (see Supplemental Information 2.2 in Multimedia Appendix 2). Therefore, all-distilroberta-v1 was selected as the optimal embedding model for subsequent tool development and deployment.

Textbox 1. **Evaluation themes and representative queries used for CanRisk-RAG development.**

**Model recommendation tasks based on cancer type**

- Breast cancer
- Lung cancer
- Prostate cancer

**Model recommendation tasks based on model type**

- Logistic regression
- Support vector machine
- Neural network

**Model recommendation tasks based on predictive factors**

- Age, gene mutation, and family history
- PSA level, digital rectal examination, and prior biopsy
- Sex, smoking status, emphysema, and pulmonary nodule

**Comprehensive model recommendation tasks**

- Gastric cancer; logistic regression; predictive factors: age, family history, and BMI
- Colorectal cancer; Cox proportional hazards regression; predictive factors: prior biopsy, alcohol intake, and height
- Bladder cancer; nomogram models; predictive factors: race, gene mutation, smoking status, and education

#### Multifactor Ranking Algorithm

To enhance credibility and practical utility, 3 additional factors were integrated into the ranking: the JIF as an indicator of publication quality [31], the AUC as a measure of model performance, and publication recency to emphasize timely contributions. The AUC was treated as a continuous metric using a smoothed compression function, while JIF values were normalized through a log-smoothing transformation to mitigate right-skewed distributions [37]. The publication year was linearly normalized to yield a continuous timeliness score. These components were then combined into a unified weighted scoring function (Equation 1).


(1) $$\begin{array}{rl} \text{Total score}= & \alpha \times \text{similarity score}+\beta \times \text{journal impact factor score}+\\ \gamma \times \text{AUC score}+\delta \times \text{timeliness score} \end{array}$$

#### LLM-Based Literature Summarization

To support user interpretation, CanRisk-RAG incorporates an automated summarization component that synthesizes the content of retrieved studies into concise, coherent overviews. By integrating information across multiple abstracts, the system highlights key findings and methodological features, enabling users to rapidly grasp the essential contributions of relevant models.

### Assessment Experiments

#### Benchmarking and Evaluation Design

To optimize the performance of CanRisk-RAG, we conducted a comprehensive comparative evaluation using 12 representative queries spanning 4 thematic areas, including cancer types, modeling approaches, predictive factors, and integrated multicomponent queries. Each theme was further divided into subthemes to reflect major research directions in cancer risk prediction [38-47] (see Textbox 1). Eight domain experts, including 3 clinical cancer researchers and 5 biomedical informatics specialists, independently assessed the outputs for all themes, with each query evaluated by 2 experts. In parallel, multiple ablation experiments on the training dataset (Table 1) examining alternative weighting schemes enabled the optimization of the final ranking configuration, ensuring balanced and robust system performance.

**Table 1.** Weight configurations for multifactor ranking algorithm used in ablation experiments.

Group	α (Similarity)	β (JIF)^a^	γ (AUC)^b^	δ (Timeliness)
G1 (Proposed)	0.85	0.05	0.05	0.05
G2 (Similarity only)	1	0	0	0
G3 (AUC+)	0.7	0.05	0.2	0.05
G4 (Recency+)	0.7	0.05	0.05	0.2
G5 (JIF+)	0.7	0.2	0.05	0.05
G6 (Similarity + Recency)	0.85	0	0	0.15
G7 (Similarity + AUC)	0.85	0	0.15	0
G8 (Similarity + JIF)	0.85	0.15	0	0
G9 (Similarity + Recency + AUC)	0.8	0	0.1	0.1
G10 (Similarity + Recency + JIF)	0.8	0.1	0	0.1
G11 (Similarity + AUC + JIF)	0.8	0.1	0.1	0

a JIF: journal impact factor.

b AUC: area under the curve.

At the application level, CanRisk-RAG was benchmarked against PubMed, ChatGPT-4o [48], ScholarAI [49], and Gemini 1.5 Flash [50], each accessed through their respective application programming interfaces under standardized query conditions. Prompts were standardized across systems to maintain fairness in comparison (see Supplemental Information 1.8 in Multimedia Appendix 1).

This stage was evaluated by domain experts. Performance was scored on 5 key dimensions: relevance, reliability, authenticity, data completeness, and consistency (0‐10 scale). Table 2 summarizes the evaluation types, metrics, and definitions used to assess the 5 applications, including CanRisk-RAG (see Supplemental Information 1.9 in Multimedia Appendix 1 for the scoring criteria of the 5 evaluation metrics).

**Table 2.** Evaluation metrics and definitions used for model recommendation and tool usability assessment.

Evaluation metrics type and metric names		Definition
Model recommendation performance evaluation		
	Relevance	The purpose of the relevance assessment is to determine the extent to which the recommended models match the user's specified criteria.
	Reliability	The reliability assessment examines the authority of the sources where the models were published and evaluates the performance of the models themselves.
	Authenticity	Authenticity checks whether the sources of the models are genuine and can be verified in various journal databases.
	Data completeness	Data completeness evaluates the thoroughness of the model information provided by each application, including metadata, cancer type, modeling type, model performance, predictive factors, and more.
	Consistency	Consistency evaluation measures how consistently the model recommendation results align when the same search conditions or prompts are used in 2 separate instances.
Tool usability evaluation		
	User goal achievement	This metric evaluates whether the tool meets users’ specific needs and expectations under open-ended, self-defined query scenarios. Participants independently formulated queries based on their own research or clinical information needs. The score reflects the extent to which users were able to successfully locate relevant cancer risk prediction models that satisfied their personalized model recommendation requirements.
	Operative accessibility	Operative accessibility refers to the overall ease of using the tool and encompasses aspects such as deployment complexity, interface design, navigation intuitiveness, and loading speed. A user-friendly tool should minimize operational complexity and cognitive load while offering clearly structured content, intuitive workflows, and comprehensive help pages.

#### Validation and Usability

To prevent data leakage between hyperparameter tuning and final performance assessment, we strictly separated the datasets used for weight optimization and for comparative evaluation. Weight optimization and ablation analyses were conducted solely on the development dataset, whereas the final benchmarking against baseline applications (PubMed, ChatGPT-4o, ScholarAI, and Gemini 1.5 Flash) was performed on an independent, held-out evaluation set. This evaluation set consisted of 20 carefully designed queries (Supplemental Information 1.10 in Multimedia Appendix 1) covering previously unseen cancer types, modeling approaches, and multifactorial scenarios that jointly specified cancer type, model type, and predictive factors. Recommendation outputs were independently assessed by eight domain experts using the predefined five evaluation dimensions.

In addition, usability was assessed by 10 potential end users, including clinicians and biomedical informatics researchers, based on 2 criteria: goal achievement and operative accessibility (see Supplemental Information 1.11 in Multimedia Appendix 1 for the scoring criteria). Inclusion criteria of potential end users required participants to be able to independently complete retrieval tasks. To mitigate potential conflict-of-interest bias, individuals involved in system development or dataset curation were excluded from usability scoring. None of the evaluators were members of the development team.

This multiperspective evaluation enabled a systematic assessment of CanRisk-RAG’s retrieval quality and practical applicability in comparison to existing systems. Table 2 summarizes the evaluation types, metrics, and definitions used for the assessment of CanRisk-RAG.

### Statistical Analysis

The evaluation was conducted in 2 phases: first, the embedding models were assessed, then the 5 applications were evaluated. Descriptive statistics on the scores were given by the evaluators, including the mean and SD. To determine the consistency among evaluators for every metric assessed, we conducted a 2-way intraclass correlation coefficient analysis.

For the evaluation of different applications, the Shapiro-Wilk test was employed to determine whether the scores assigned by different evaluators for a specific metric followed a normal distribution. Due to the departure of the scores from normality, the Mann-Whitney *U* test was selected to compare the overall performance scores. To explore variations in performance across the five applications and various subthemes, the Kruskal-Wallis rank-sum test was employed. When significant differences were identified by the Kruskal-Wallis test, Dunn post hoc multiple comparisons test was used to evaluate the performance of the different baselines in relation to CanRisk-RAG. Cliff delta was reported for all pairwise comparisons. Multiplicity control across post hoc tests was handled using Benjamini–Hochberg adjustments (*α*=.05). A significance level of *P*<.05 was applied to all statistical tests. All statistical tests were performed using Python (version 3.12).

### Ethical Considerations

This study did not involve human or animal subjects; therefore, an ethics review board assessment was not required in accordance with institutional and national guidelines of Sichuan University. All analyses were conducted on publicly available, nonidentifiable data, consistent with regional research ethics policies [51].

## Results

### Tool Interface

**Figure 3.**
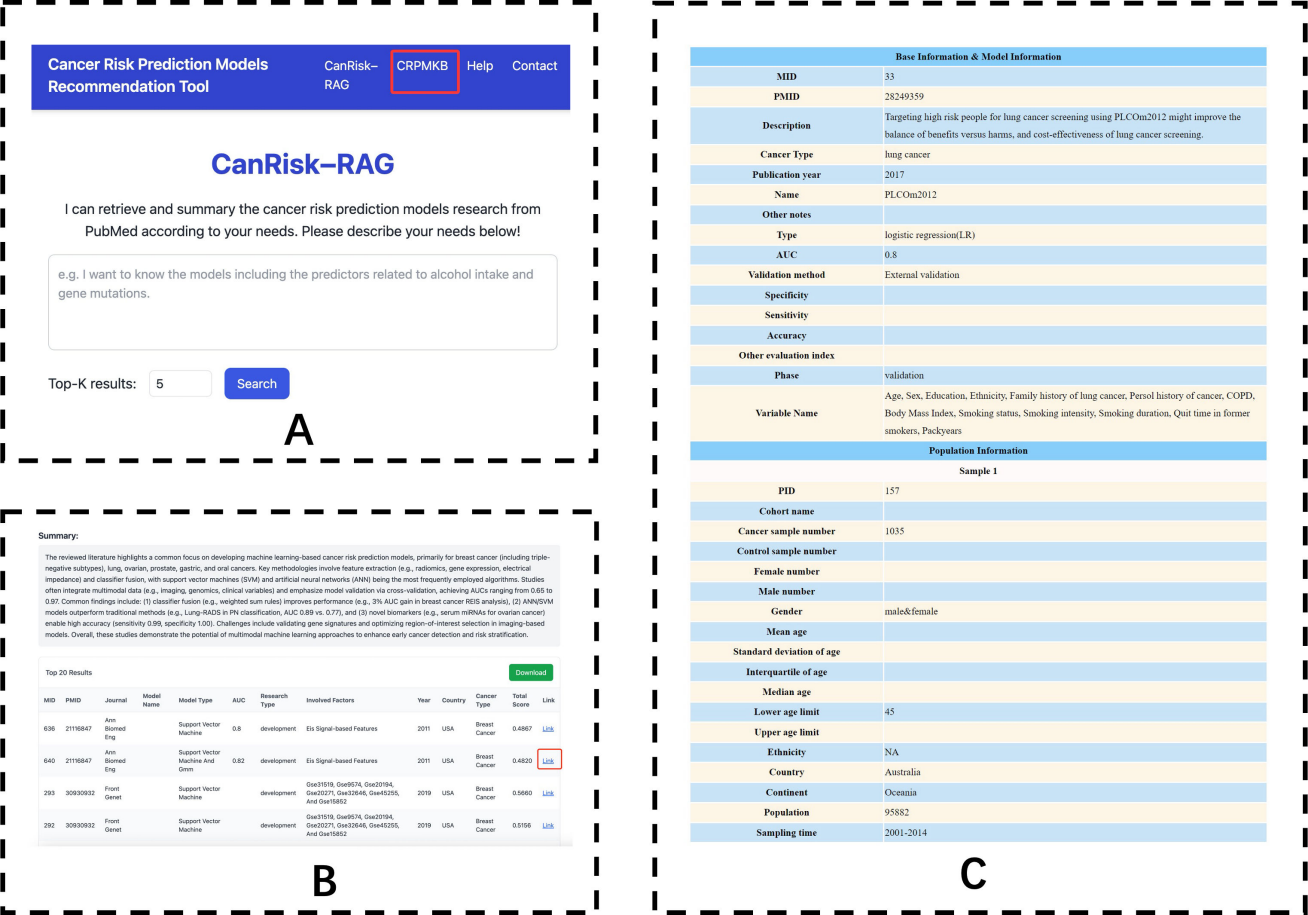
CanRisk-RAG tool interfaces. (A) Homepage query form. (B) Recommendation results page. (C) Detailed model information page.

As shown in Figure 3, the homepage of CanRisk-RAG features an interactive input form (Figure 3A) that allows users to specify any retrieval criteria related to cancer risk prediction models. Users may define the desired number of recommended results through a customizable selection option. Additionally, users can directly access the data source associated with the homepage by clicking the “CRPMKB” link in the main navigation bar. After entering the desired model criteria in the input field and clicking the “Search” button, the system displays the results page (Figure 3B), which presents a list of recommended models retrieved based on the user’s specified conditions. In the table result, each recommended model is presented with detailed information, including attributes such as MID, PMID, Journal, Model Name, Model Type, AUC, Research Type, Involved Factors, Year, Country, Cancer Type, Total Score. Each model entry is hyperlinked to its corresponding detailed record (Figure 3C). Furthermore, for all recommended models, the tool automatically generates a summary of the corresponding research abstract (Figure 3B), providing a concise overview of these studies to support rapid understanding and comparison.

### Model Recommendation Evaluation Results

#### Weight Configuration

We systematically evaluated eleven alternative weighting configurations (G1-G11) that varied the relative contributions of similarity (α), JIF (β), model AUC (γ), and publication timeliness (δ) (Table 3). Each configuration was tested on the same dataset of representative queries, and the average scores for relevance, reliability, and total performance were calculated. A nonparametric Kruskal–Wallis *H* test revealed a statistically significant overall difference among the eleven configurations (*H*=47.916, *P*<6.430 × 10⁻⁷, see Supplemental Information 2.3 in Multimedia Appendix 2).

The proposed configuration (G1: *α*=.85, *β*=.05, *γ*=0.05, *δ*=0.05) achieved the highest overall score, significantly outperforming variants such as SimOnly and Recency+ (*P*_Holm_<.01). G1 consistently yielded higher scores across all configurations, demonstrating a robust performance on relevance and reliability.

**Table 3.** Performance comparison of different weighting configurations (mean [SD] for relevance, reliability, and total scores).

Group	Avg_Relevance, mean (SD)	Avg_Reliability, mean (D)	Avg_Total, mean (SD)
G1 (Proposed)	9.50 (0.78)	8.88 (0.85)	18.38 (1.44)
G8 (Similarity + JIF^a^)	8.50 (0.66)	9.21 (0.78)	17.71 (1.16)
G7 (Similarity + AUC^b^)	8.92 (0.97)	8.75 (0.85)	17.67 (1.55)
G6 (Similarity + Recency)	9.25 (0.74)	8.04 (0.81)	17.29 (1.12)
G3 (AUC+)	7.96 (0.96)	9.25 (0.90)	17.21 (1.59)
G5 (JIF+)	8.17 (0.92)	8.67 (0.96)	16.83 (1.37)
G10 (Similarity + Recency + JIF)	8.08 (0.78)	8.25 (0.79)	16.33 (0.87)
G9 (Similarity + Recency + AUC)	8.17 (1.05)	8.08 (0.93)	16.25 (1.51)
G11 (Similarity + AUC + JIF)	7.83 (0.64)	7.92 (0.72)	15.75 (0.79)
G2 (Similarity only)	9.42 (0.65)	5.46 (0.72)	14.88 (1.08)
G4 (Recency+)	7.33 (0.96)	7.08 (0.72)	14.42 (1.06)

a JIF: journal impact factor.

b AUC: area under the curve.

#### Performance Comparison of Applications

In the comparative evaluation across 5 applications, CanRisk-RAG consistently achieved the highest relevance and reliability across all 4 themes of the validation set (Table 4, see Supplemental Information 2.4 and 2.5 in Multimedia Appendix 2 for detailed statistics), delivering markedly superior retrieval precision compared with existing applications. For relevance, the average score was 8.30 (SD 0.59; 95% CI 8.02-8.58), indicating that the retrieved models closely matched the intended cancer types, modeling approaches, and predictive factors specified in the queries. In terms of reliability, the average score reached 7.62 (SD 0.76; 95% CI 7.27-7.98), reflecting strong consistency with authoritative and well-validated literature sources. These results confirm that, when applied to independent validation themes, CanRisk-RAG maintains robust generalization and stable interpretability, effectively extending to unseen data while preserving retrieval accuracy and quality consistency, thereby underscoring its scalability and real-world applicability. PubMed showed reasonable accuracy for straightforward cancer-type queries but struggled when tasks involved modeling approaches or specific predictive factors, often returning incomplete or no usable results. Scholar AI performed moderately in simpler recommendation tasks, but its accuracy dropped sharply for more complex, integrative queries. ChatGPT-4o occasionally retrieved relevant cancer-type models but frequently missed methodological or variable-level details. Gemini 1.5 Flash repeatedly failed to interpret user intent or generate sufficient recommendations, underscoring its limited capability for structured retrieval.

**Table 4.** Comparative evaluation of relevance and reliability on the test set across 5 applications.

Applications	Relevance, mean (SD)	*P*_bh_ ^a^	Reliability, mean (SD)	*P*_bh_
Topic 1				
CanRisk-RAG	8.70 (0.45)	—^b^	8.10 (0.55)	—
ChatGPT-4o	5.90 (1.24)	<.05	3.50 (0.35)	<.05
Gemini 1.5 Flash	2.40 (0.42)	<.05	1.80 (0.57)	<.05
PubMed	5.70 (0.97)	<.05	7.20 (1.04)	ns^c^
ScholarAI	7.90 (1.02)	ns	6.60 (0.96)	ns
Topic 2				
CanRisk-RAG	8.50 (0.50)	—	7.80 (0.76)	—
ChatGPT-4o	1.30 (0.84)	<.05	1.00 (0.35)	<.05
Gemini 1.5 Flash	1.20 (0.45)	<.05	1.80 (0.27)	<.05
PubMed	2.40 (1.14)	<.05	6.50 (1.00)	ns
ScholarAI	6.10 (0.74)	<.05	5.20 (1.30)	<.05
Topic 3				
CanRisk-RAG	8.40 (0.42)	—	7.20 (0.76)	—
ChatGPT-4o	1.80 (0.27)	<.05	1.10 (0.42)	<.05
Gemini 1.5 Flash	1.10 (0.42)	<.05	0.90 (0.55)	<.05
PubMed	1.20 (0.57)	<.05	5.60 (2.90)	ns
ScholarAI	5.10 (0.89)	<.05	4.70 (1.82)	ns
Topic 4				
CanRisk-RAG	7.60 (0.42)	—	7.40 (0.82)	—
ChatGPT-4o	2.20 (0.57)	<.05	1.10 (0.55)	<.05
Gemini 1.5 Flash	1.90 (0.22)	<.05	1.70 (0.57)	<.05
PubMed	0.10 (0.22)	<.05	0.40 (0.42)	<.05
ScholarAI	1.80 (1.15)	<.05	1.20 (1.04)	<.05
Average score				
CanRisk-RAG	8.30 (0.59)	—	7.62 (0.76)	—
ChatGPT-4o	2.80 (2.01)	<.05	1.68 (1.15)	<.05
Gemini 1.5 Flash	1.65 (0.65)	<.05	1.55 (0.60)	<.05
PubMed	2.35 (2.28)	<.05	4.92 (3.13)	<.05
ScholarAI	5.22 (2.44)	<.05	4.42 (2.37)	<.05

a *P*_bh_: Benjamini–Hochberg adjusted significance levels, representing statistical comparisons against the corresponding CanRisk-RAG scores within the same topic or in the overall average.

b Not applicable.

c Not significant.

**Figure 4.**
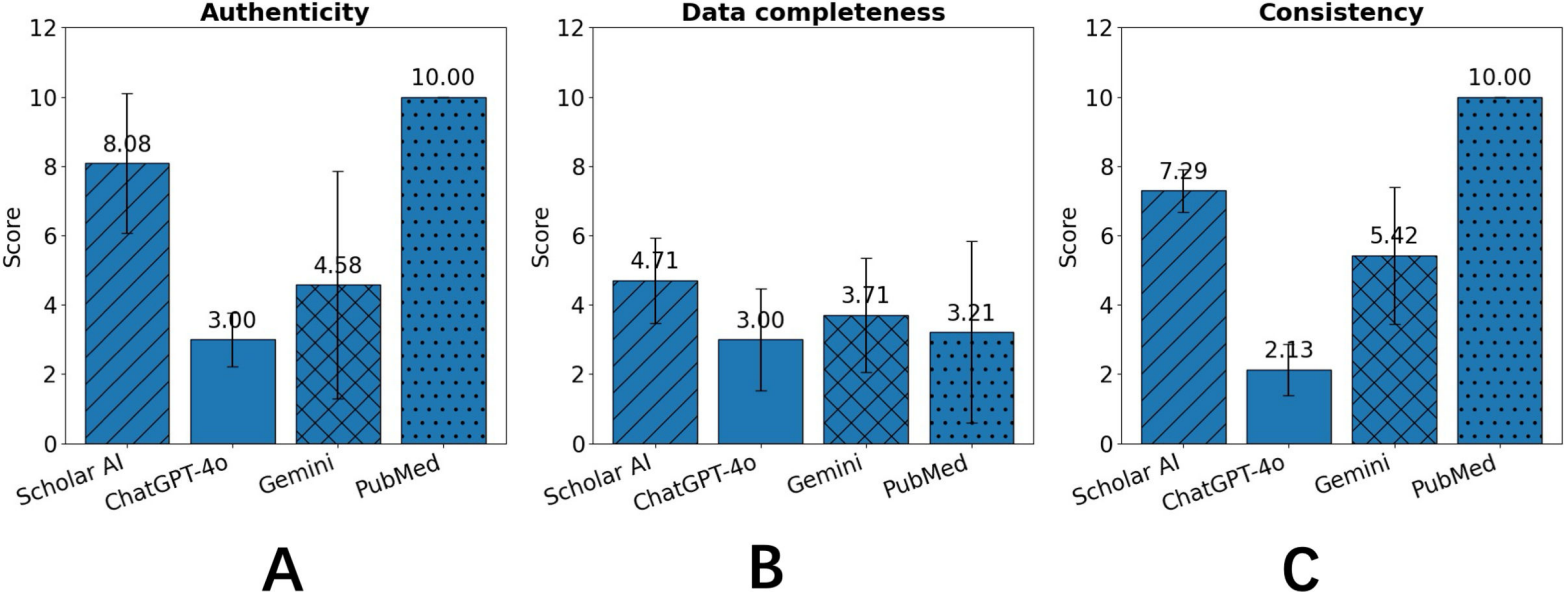
Comparative evaluation of authenticity, data completeness, and consistency among 4 baseline applications in cancer risk prediction model recommendation tasks.

As a structured and knowledge-grounded recommendation system, CanRisk-RAG achieved high performance in authenticity, data completeness, and consistency, and was therefore used as the benchmark for evaluating other applications (Figure 4). PubMed, while fully reliable in authenticity and consistency due to its literature-based retrieval, showed only moderate data completeness, particularly failing in the comprehensive recommendation task. The 3 LLM-based tools, Scholar AI, ChatGPT-4o, and Gemini 1.5 Flash, performed less reliably overall and often generated incomplete or inconsistent metadata (see Supplemental Information 2.6 in Multimedia Appendix 2). Scholar AI displayed partial strength across themes but still suffered from missing model details, while Gemini 1.5 Flash showed some consistency in recommending models based on cancer types and modeling approaches yet frequently fabricated or cited nonscientific sources such as blogs or online news (see relevant examples in Supplemental Informations 1.12 and 1.13 in Multimedia Appendix 1).

Overall, these findings demonstrate that CanRisk-RAG effectively bridges structured domain knowledge with LLM-driven semantic reasoning, achieving both high retrieval precision and reproducibility. Across all evaluation metrics, CanRisk-RAG consistently outperformed PubMed, Scholar AI, ChatGPT-4o, and Gemini 1.5 Flash, attaining markedly higher relevance and reliability alongside near-perfect scores in authenticity, data completeness, and consistency. In contrast, the baseline systems frequently struggled with complex, multifactorial queries, often returning incomplete, inconsistent, or fabricated information.

### Usability Evaluation Results

The usability scores are summarized in Supplemental Information 14 in Multimedia Appendix 1. On a 3-point scale, CanRisk-RAG achieved a mean score of 2.7 (SD 0.48) for user goal achievement, indicating that the system was generally effective in meeting users’ personalized retrieval needs. In addition, the tool demonstrated good operative accessibility, with an average score of 2.5 (SD 0.53), suggesting that users found the interface and overall operation intuitive and easy to use. Taken together, these results indicate that CanRisk-RAG is user-friendly, enabling most users to accomplish their intended tasks efficiently with minimal operational burden.

## Discussion

### Principal Findings

The results highlight CanRisk-RAG’s clear advantages in recommending cancer risk prediction models, outperforming general-purpose LLM tools and traditional systems like PubMed. By combining an organized knowledge base, which provides rich metadata including cancer types, modeling methods, and performance metrics with a RAG framework, CanRisk-RAG enables high-quality personalized recommendations and advances beyond keyword-based retrieval toward expert-level semantic recommendation. Unlike ChatGPT-4o, Scholar AI, and Gemini 1.5 Flash, which often hallucinate content or misinterpret domain-specific queries, CanRisk-RAG delivers accurate recommendations with structured information grounded in validated literature. It should be noted that as a peer-reviewed, specialized biomedical literature database, PubMed achieved near-perfect scores in authenticity and consistency, which should be interpreted as a reference baseline rather than an algorithmic achievement. While PubMed inherently ensures authenticity through direct literature indexing, it lacks the semantic parsing capabilities required for complex, multifactorial queries, thereby underscoring the value of domain-specific systems that integrate structured knowledge with semantic understanding. It should also be acknowledged that a degree of circularity exists between the system design of CanRisk-RAG and the reliability evaluation. Specifically, JIF is incorporated as a low-weight auxiliary signal in the ranking algorithm, while the reliability metric used in expert evaluation partially reflects publication in authoritative journals. Consequently, the high reliability scores achieved by CanRisk-RAG should be partly interpreted as evidence that the system adheres to its predefined design principles, namely prioritizing models supported by established, higher-reputation peer-reviewed sources, rather than being overinterpreted as direct indicators of clinical effectiveness.

The system adopts a dual-embedding strategy that fuses tag-level semantic representations with embeddings of the original user query, allowing it to jointly capture structured domain constraints and broader user intent. This design enables effective semantic matching even in the absence of exact keyword overlap. Building on this representation, a multi-factor ranking algorithm integrates semantic similarity with complementary quality indicators—including JIF, model AUC, and publication recency—to enhance the credibility and practical usefulness of the recommended models. This is particularly beneficial for model comparison and systematic review scenarios, where both relevance and evidential strength are critical. In addition, our tag-extraction evaluation demonstrates that DeepSeek-V3 achieves high overall accuracy (micro-*F*_1_=0.994), with only two omission cases observed in the 40-query test set. These rare errors were primarily attributable to implicit semantic descriptors or typographical issues rather than systematic extraction failures. Importantly, the fused embedding framework provides partial robustness to such upstream imperfections, as the original-query embedding channel can still encode missing concepts even when a structured tag is not completely extracted.

With respect to the comparative evaluation strategy, baseline LLM systems, namely ChatGPT-4o, ScholarAI, and Gemini 1.5 Flash, were evaluated using static zero-shot prompts to ensure standardized and reproducible conditions across tools. Although this design choice reduces prompt engineering bias and enhances fairness, it may underestimate the upper bound performance of general purpose LLMs, which can benefit from chain of thought prompting, multiround clarification, or interactive refinement. However, incorporating adaptive and tool-specific prompt optimization would compromise reproducibility and comparability, as performance would then depend on prompt engineering expertise rather than intrinsic system capability. Importantly, CanRisk-RAG does not rely on iterative user clarification. Instead, it embeds domain knowledge and semantic constraints directly into the retrieval pipeline, reflecting a distinct design philosophy that shifts cognitive and procedural burden from the user to the system. This property is particularly important in clinical and translational research settings. Moreover, unlike interactive LLM-based tools, CanRisk-RAG produces deterministic and reproducible outputs for identical inputs, which is essential for evidence-based model discovery and systematic research workflows.

Although the superior performance of CanRisk-RAG highlights the advantage of integrating structured domain knowledge with retrieval-augmented generation, its effectiveness largely depends on the built-in knowledge base, which supplies high-quality, expert-verified model information. Consequently, the “extensibility” of the framework depends on the sustainable expansion of such constructed resources. To address this scalability challenge, we developed a semiautomated update pipeline that combines automated literature retrieval, rule-based metadata extraction, and LLM-assisted knowledge extraction in a workflow [52]. This tool substantially improves data update efficiency while reducing manual workload. Nevertheless, essential human review and expert supplementation are retained to ensure the accuracy of curated content. The knowledge base is planned to be updated every six months to maintain both recency and reliability.

In addition, although currently focused on cancer risk prediction models, the CanRisk-RAG framework is adaptable to other disease domains and scenarios, including prognosis and treatment response models, underscoring its potential as a universal platform for the discovery and application of clinical prediction models.

### Limitations

Despite the encouraging performance of CanRisk-RAG, several limitations should be acknowledged.

First, although CanRisk-RAG offers enhanced flexibility and effective retrieval, its performance is inevitably shaped by multiple structured layers of uncertainty spanning internal (algorithmic) and external (evidence-base) factors. Internally, reliance on semantic similarity may reduce field-level specificity; LLM-based tag extraction can over-generalize factors (eg, interpreting “family history of breast cancer” as “family history” or generalizing a specific genetic factor such as BRCA1/2 mutation into a broad category like “genetic risks”) and changes in multi-factor weights (eg, raising “Recency” weight from 0.05 to 0.15) can alter rankings. These factors introduce algorithmic uncertainty, which we partially mitigated through ablation experiments and iterative weighting optimization. Externally, heterogeneity in study design, sample size, population, and reporting limits comparability and generalizability. For instance, lung cancer survival models from small regional cohorts may not generalize to multi-institutional datasets, and models trained on East Asian populations may underperform in Western populations due to demographic and healthcare-system differences. Even established models (eg, Gail) exhibit cohort-dependent AUC and calibration, highlighting cross-dataset variability as an important uncertainty layer. Future work will incorporate domain adaptation and uncertainty quantification methods to reduce these effects.

Second, the current ranking strategy relies on a limited set of quality-related indicators. JIF is included as a low-weight auxiliary signal and is normalized to mitigate its influence; however, it remains a coarse, venue-level metric that does not directly reflect study-level methodological rigor. Important indicators such as sample size, calibration reporting, and the extent of external validation are not yet uniformly available across the curated literature and therefore could not be systematically incorporated as first-class ranking features in the present version. As a result, residual bias may be introduced when finer-grained quality signals are missing or inconsistently reported, even though JIF is used only to refine the ordering among semantically similar candidates. In addition, the ranking strategy incorporates reported AUC values as a quantitative indicator of model performance without explicitly differentiating or weighting AUCs derived from model development versus external validation stages. This design choice may inadvertently favor development studies that report optimistic performance over rigorously validated models with stronger generalizability. Future versions of CanRisk-RAG will aim to integrate validation-aware performance stratification and explicit study-level quality indicators, such as cohort size, validation design, and calibration metrics, to better balance apparent accuracy with real-world applicability and progressively reduce reliance on venue-level signals.

Third, the present study relied on a single LLM (DeepSeek-V3) for semantic tag extraction and literature summarization. While this design choice ensured internal consistency and reproducibility across experiments, it inevitably limited robustness to model-specific variability and potential biases inherent to a single LLM. In addition, baseline comparisons with general-purpose LLMs were conducted under a static zero-shot prompting setting without access to external retrieval, web browsing, or tool invocation capabilities. As a result, these baselines were constrained to parametric-only knowledge, which may underestimate their performance in more interactive or retrieval-augmented configurations. In this context, the evaluation primarily highlights the hallucination risks and structural limitations of ungrounded LLM responses when external knowledge is unavailable, rather than serving as an exhaustive benchmark of the full capabilities of modern LLM ecosystems. Future work will extend this evaluation framework by incorporating multi-model ensemble strategies for tag extraction and summarization to enhance robustness and stability. In parallel, more comprehensive comparisons will be conducted against LLM-based systems equipped with web browsing, tool use, or fully integrated RAG workflows, including multi-turn and interactive prompting strategies.

Fourth, expert scoring was performed manually, introducing subjectivity despite inter-rater consistency checks. Future studies will incorporate automated evaluation methods, such as rule-based matching and semantic alignment metrics, to improve reproducibility and reduce dependence on manual assessments.

Fifth, although CanRisk-RAG includes an LLM-based literature summarization module, the current evaluation is primarily centered on retrieval and recommendation performance. The adopted metrics assess the relevance and reliability of retrieved models and metadata but do not explicitly quantify the factual accuracy or hallucination rate of the generated summaries. The summarization component is intended to serve as an assistive tool for facilitating user comprehension and synthesizing key information from retrieved literature, rather than to provide precise quantitative data; future work will incorporate dedicated evaluation protocols to systematically assess the quality and faithfulness of the generative component.

Finally, a key limitation of the present study is that the CanRisk-RAG knowledge base was constructed using literature retrieved prior to December 2022, and therefore does not include cancer risk prediction models published from 2023 onward. This temporal constraint may temporarily place CanRisk-RAG at a relative disadvantage when compared with contemporary LLMs that potentially have access to more recent biomedical literature. Beyond general concerns regarding data currency, this cutoff has specific implications for users seeking state-of-the-art computational or deep learning–based cancer risk prediction models, as recent years have witnessed a rapid proliferation of AI-driven approaches incorporating deep neural networks and multimodal data integration. As a result, the current version of CanRisk-RAG may be less suitable for users whose primary objective is to identify the most recent model architectures. To address this limitation and enhance temporal coverage, we have developed a semiautomated update pipeline that integrates automated literature retrieval, rule-based metadata extraction, and LLM-assisted curation, while retaining expert review to ensure data quality. The knowledge base is planned to be updated on a semiannual basis. Future evaluations will incorporate newly published models to assess how expanded temporal coverage influences retrieval performance, and to enable more temporally aligned comparisons with evolving LLM-based systems, particularly in the context of rapidly advancing AI-driven cancer risk modeling.

### Conclusion

This study introduced CanRisk-RAG, a knowledge-driven retrieval framework for cancer risk prediction models that integrates structured knowledge with semantic reasoning. Through systematic evaluation, CanRisk-RAG demonstrated superior performance in relevance, reliability, and data consistency compared with existing retrieval systems. These results indicate that combining structured knowledge representation with LLM-based reasoning can substantially improve the precision and transparency of model discovery in biomedical research, supporting reproducible and evidence-based applications in precision oncology.

## Supplementary material

10.2196/78519 Multimedia Appendix 1 Supplementary methods and details.

10.2196/78519 Multimedia Appendix 2 Raw scores of experiments.

## Abbreviations

AUC: area under the curve
JIF: journal impact factor
LLM: large language model
MID: model ID
RAG: retrieval-augmented generation
