# Supplementary material for: Knowledge-Guided Explainable Recommendation Tool for Cancer Risk Prediction Models Using Retrieval-Augmented Large Language Models: Development and Validation Study
Source: JMIR Med Inform. 2026 Mar 9;14:e78519. doi: 10.2196/78519 (PMC12978927; doi:10.2196/78519)
Supplement: Multimedia Appendix 1 [file medinform-v14-e78519-s001.docx]

Supplemental information 1.1 Methods of constructing CanRisk-RAG.

1. Data Segmentation and Vectorization

We constructed a FAISS-based vector database using CanRisk-RAG’s data. To retain the most relevant model information, each entry in the knowledge base was encoded into the vector database using key fields, including *Cancer_Type*, *Model_Type*, *Research_Type*, *Involved_Factors*, *Area*, and *Abstract*. Specifically, the fields of *Cancer_Type*, *Model_Type*, *Research_Type*, *Involved_Factors*, and *Country*, were concatenated into a composite string—referred to as the *tag_string*—representing the core semantic features of each model. This *tag_string* was then encoded using a pre-trained SentenceTransformer model to generate *tag_embeddings*, capturing the semantic meaning of the key model information. Likewise, the *Abstract* field of each model was independently encoded into *abstract_embeddings*, which capture supplementary contextual information beyond the structured tags. Together, these semantic vectors represent the essential content of each model entry. It should be noted that models with identical names but different validation cohorts are treated as distinct entries, reflecting their data-specific characteristics.

1. Tag Extraction via LLM for Enhanced Semantic Matching

We developed a front-end interface for CanRisk-RAG that enables users to submit free-text queries describing their models of interest, with the option to specify the number of recommended results. Upon submission, the input is processed by a large language model, for which we selected DeepSeek-V3(API version 2025-10, temperature = 0.2, top-p = 0.9, output limit = 2,048 tokens). Rather than directly matching user input against the vector database, the system first extracts semantic tags to capture user intent based on predefined fields. A tailored prompt guides the LLM to identify essential attributes, including Model_Type, Research_Type, Cancer_Type, Involved_Factors, and Country. This process leverages the natural language understanding capabilities of LLMs to transform unstructured queries into structured semantic representations, thereby enabling efficient retrieval. The extracted information is returned in JSON format, subsequently cleaned and parsed to generate structured tag combinations for downstream semantic matching. To evaluate whether DeepSeek-V3 could reliably perform semantic reasoning and field extraction, we designed 40 test queries assessed independently by two researchers, with discrepancies adjudicated through cross-validation by a third expert to ensure consistency of the results (see Supplemental Information 1.6).

1. Embedding Models Selections

To prioritize relevant models, CanRisk-RAG employs a ranking strategy primarily driven by semantic similarity. User queries are semantically aligned with database entries through embedding-based similarity, which captures both structured tags and contextual meaning. The similarity score serves as the main determinant, reflecting the degree of correspondence between user intent and model characteristics.First, **SentenceTransformer** is employed to generate embeddings for both the user-input tag string and the original query text, respectively. SentenceTransformer is a method based on pre-trained Transformer models, designed to encode sentences or text segments into high-dimensional semantic vectors to support semantic similarity computation and efficient text retrieval tasks.

A weighted fusion strategy was adopted, assigning a weight of **0.8** to the tag embedding and **0.2** to the query text embedding. This hybrid approach allows the final semantic vector to comprehensively represent the user's retrieval intent. Cosine similarity is used as the metric to quantify the semantic alignment between the query and FAISS-indexed entries.

Specifically, for semantic retrieval, two parallel similarity channels were constructed. First, the structured tag string extracted from the user query was encoded into a *query_tag_embedding* and compared against the database *tag_embeddings* using cosine similarity, yielding a tag-level similarity score (*sim_tag*). In parallel, the original free-text user query was independently encoded into a *query_text_embedding* and compared against the database *abstract_embeddings*, producing a complementary text-level similarity score (*sim_text*). The final semantic similarity score was computed as a weighted linear combination of these two channels:

Final similarity = 0.8 × sim_tag + 0.2 × sim_text

(eq.1)

To determine which embedding model achieved the best semantic representation performance, we evaluated four widely used sentence-embedding architectures—all-MiniLM-L6-v2, all-MiniLM-L12-v2, all-mpnet-base-v2, and all-distilroberta-v1—on the training dataset (Table 1 in the main text). Each model’s output was assessed by two independent evaluators across 12 predefined recommendation queries derived from four thematic categories. For each model, average relevance and reliability scores were calculated. Because the Shapiro–Wilk test indicated non-normal distributions, non-parametric tests were applied for subsequent analysis (see Supplemental Information 2.3).

Inter-rater consistency was high for both relevance (ICC[3,k] = 0.98) and reliability (ICC[3,k] = 0.888), confirming stable and consistent evaluations between raters. The Kruskal–Wallis test revealed significant overall differences among the four embedding models for both relevance (H = 11.432, p = 0.0096) and reliability (H = 9.238, p = 0.0263).

Pairwise Mann–Whitney U tests further showed that the all-distilroberta-v1 model outperformed all-MiniLM-L6-v2 in both relevance (U = 185, p = 0.0254) and reliability (U = 172.5, p = 0.0141), and similarly exceeded all-MiniLM-L12-v2 (U = 130, p = 0.0002; U = 164, p = 0.0073) and all-mpnet-base-v2 (U = 195, p = 0.0352; U = 190, p = 0.0357) in both metrics.
The mean ± SD total scores were 17.92 for all-distilroberta-v1, 16.33 for all-mpnet-base-v2, 15.75 for all-MiniLM-L12-v2, and 14.92 for all-MiniLM-L6-v2.

Taken together, these analyses indicate that all-distilroberta-v1 achieved the highest overall performance, with statistically significant advantages in both relevance and reliability metrics. Therefore, all-distilroberta-v1 was selected as the optimal embedding model for subsequent tool development and deployment.

1. Multi-Factor Ranking Algorithm

CanRisk-RAG achieves information retrieval through the integration of embedding vectors and a FAISS indexing system, coupled with a multi-criteria ranking algorithm.

In addition to semantic similarity, the ranking algorithm incorporates three other quality-related factors: **Journal Impact Factor (JIF); Area Under the Curve (AUC)**; and **Timeliness Score.**

The overall **Total Score** (Equation 1) is calculated to ensure a balanced consideration of semantic relevance, publication quality, model performance, and timeliness. The top K entries with the highest total scores are returned as the final recommendation results. For each retrieved model, CanRisk-RAG generates a table with detailed information and a direct link to its complete metadata.

$$\text{Total score=α×similarity score+β×journal impact factor }\text{score+γ×AUC}\text{ }\text{score+δ×timeliness}\text{ score}$$

(eq.2)

### JIF Score

To ensure that higher-impact journals receive proportionally higher scores while avoiding excessive emphasis on extremely high JIF values, we adopted a **monotonically increasing but saturating transformation** based on the exponential function. This normalization compresses the heavy-tail distribution while preserving the ordinal relationships between journals.

$$\mathrm{JIF}_{\mathrm{score}}=1-e^{-aX}$$

(eq.3)

where

$X$ denotes the raw JIF (the 2024 version), and

$a$ = 0.2 is a scaling coefficient controlling the saturation rate.

### AUC Score

To balance the contribution of model performance in the overall ranking, we applied a **piecewise linear normalization** to the raw AUC values. Since AUC values below 0.5 generally indicate poor model discrimination, and values above 1.0 cannot occur theoretically, we mapped the AUC range [0.5,1.0] to a normalized score within [0,1] using a clipping-based transformation:

$$\mathrm{AUC}\text{ }\mathrm{Scor}e=\mathrm{clip}(\frac{\mathrm{AUC}_{raw}-0.5}{0.5},0,1)$$

(eq.4)

This transformation ensures that models with AUC ≤ 0.5 receive a score of 0, while models with AUC = 1.0 receive a maximum score of 1. Intermediate AUC values are linearly scaled between 0 and 1. This approach provides a smooth and interpretable performance mapping that emphasizes meaningful performance differences in the mid-to-high AUC range without amplifying saturation effects at the upper bound.

### Timeliness Score

To incorporate the publication recency of each model into the ranking process, we computed a **timeliness score** that linearly normalizes the publication year into the range [0,1]. Specifically, given the publication year of a model ($\mathrm{Year}_{value}$) and the minimum and maximum years in the dataset ($\mathrm{Year}_{max}-\mathrm{Year}_{min}$​), the timeliness score is defined as:

$$\text{Timeliness Score}=\mathrm{clip}(\frac{\mathrm{Year}_{value}-\mathrm{Year}_{min}}{\mathrm{Year}_{max}-\mathrm{Year}_{min}},0,1)$$

(eq.5)

This normalization ensures that more recently published models receive higher timeliness scores, while older models receive proportionally lower values. The clipping function guarantees bounded scores between 0 and 1, preventing outliers from overly influencing the ranking. This linear approach provides a straightforward yet effective way to reflect the freshness and current relevance of each model within the recommendation framework. The value of $\mathrm{Year}_{max}$ was fixed at 2022, corresponding to the most recent year of literature included in the CanRisk-RAG knowledge base at the time of system construction and evaluation.

Finally, through iterative testing across training dataset queries and multiple ablation experiments with alternative weighting schemes (Table 2 in main text), the weighting configuration was optimized to achieve balanced and robust overall performance.

1. Comparative Experiment

To validate the performance of CanRisk-RAG, we conducted a comparative analysis across five applications, including PubMed and three LLM-based tools. To prevent data leakage, we used a strict train/development–test separation: (i) the multi-factor ranking weights were optimized only on a 12-query development set (Table 1 in the main text) via iterative ablation across candidate weight groups (Table 2 in the main text); and (ii) the final application-level benchmarking was conducted exclusively on a held-out test set of 20 queries (Supplemental Information 1.10). The held-out test set was designed to cover diverse cancer types, model types, predictive-factor constraints, and comprehensive multi-constraint queries, thereby assessing generalization beyond the development queries.

**Among the four compared applications**, the first is ChatGPT-4o (API version 2024-10, temperature = 0.7, top-p = 0.9, max output = 4,096 tokens), the most advanced version in the ChatGPT series, which offers enhanced capabilities in text processing and reasoning through powerful deep learning technology. The second is Scholar AI (API version 2024-10, with predefined temperature, top-p and max output), a tool specifically designed for academic research and scientific exploration, capable of accessing the databases of over 200 million peer-reviewed scientific articles to assist users in finding and summarizing academic research, patent information, and more. The third is Gemini (Gemini 1.5 Flash, API version 2024-10, temperature = 0.7, top-p = 0.9, max output = 4,096 tokens), Google's newest application in the field of LLMs. All three LLM-based tools were accessed via their respective APIs for testing. PubMed is a biomedical literature retrieval tool that provides access to authentic, peer-reviewed scientific publications across medicine and life sciences.

Supplemental information 1.2 The search terms of CanRisk-RAG knowledge base.


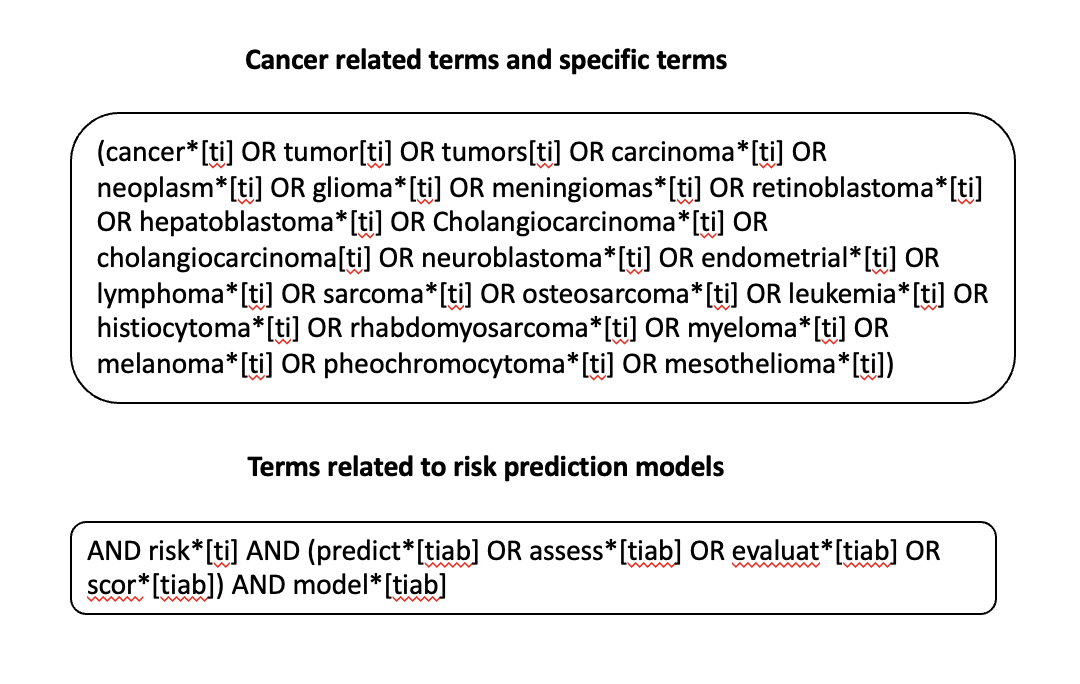


**This search term was retrieved prior to December 2022.*

Supplement information 1.3 Inclusion and Exclusion Criteria of the CanRisk-RAG data sources

The data sources mainly include multi-factor prediction models for the first occurrence of cancer of individuals, including development and validation phase.

1. The model collected should be only developed for one type of cancer instead of multiple cancers.
2. The model development or validation in the article is based on human beings.
3. Exclude research about prediction of cancer recurrence, clinical diagnosis, cancer prognosis.
4. Exclude review or meta-analysis.

Supplemental information 1.4 The Entity–Relationship (ER) diagram of CanRisk-RAG knowledge base.


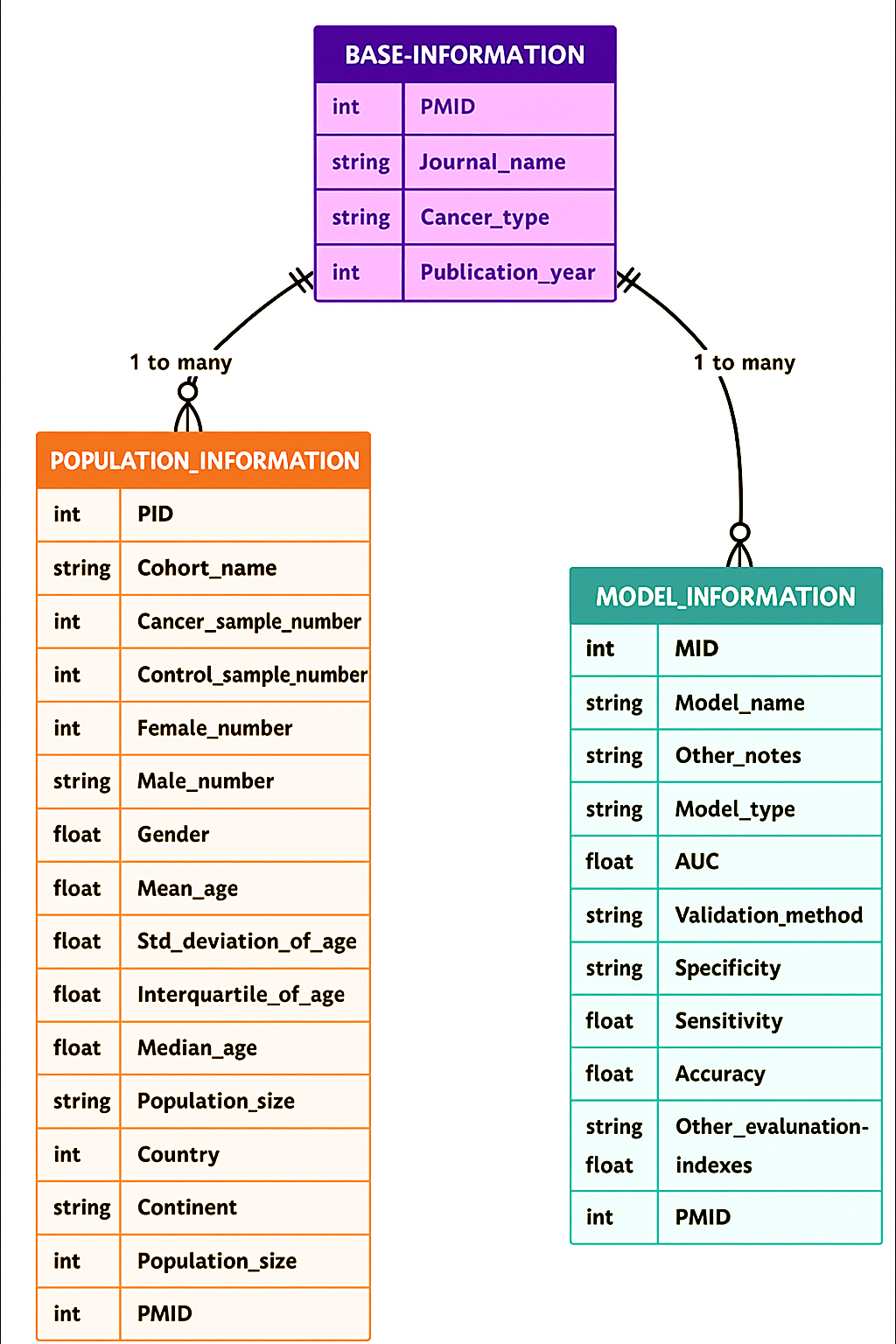


Supplemental information 1.5 Prompt Template for LLM-Based Semantic Tag Extraction

prompt = f"""

You are a biomedical information extraction assistant for cancer risk prediction model queries.

Task:

Given a user query, extract the following fields and return a single JSON object.

Output schema (must follow exactly):

{

"Cancer_Type": string | "-",

"Model_Type": string | "-",

"Research_Type": "Model Development" | "Model Validation" | "-",

"Involved_Factors": [string] | [],

"Country": string | "-",

"Original_Input": string

}

Strict rules:

1) Do NOT hallucinate. Only extract what is explicitly stated or unambiguously implied.

- If the user does NOT specify a cancer type, output "Cancer_Type": "-".

- If the user does NOT specify a model type, output "Model_Type": "-".

- If the user does NOT specify country, output them as "-".

2) Model_Type: When extracting the model type, prefer the full formal name instead of abbreviations whenever it can be unambiguously inferred from the input (e.g., use Cox Proportional Hazards Regression rather than Cox).

3) Research_Type:

- If the query includes "validation", "validated", "external validation", "validation study", "independent cohort", set Research_Type = "Model Validation".

- Otherwise set Research_Type = "Model Development".

- If even this cannot be inferred, use "-".

4) Involved_Factors:

- Extract all risk/predictor variables mentioned (e.g., age, BMI, PSA, smoking status, pack-years, BRCA1/2, HPV, CA125, AFP, CA19-9, cirrhosis, imaging features, nodule characteristics, etc.).

- Keep factor names concise but faithful; do not merge unrelated factors.

- If none are mentioned, return an empty list [].

5) Preserve Original_Input exactly (including typos).

6) Try to correct any possible typos

Now extract from the following user query:

Text:

{user_input}

"""

Supplement information 1.6 The evaluation set of the tag extraction tasks for DeepSeek-V3.

| # | User Query | Gold Tags (True Labels) | DeepSeek-V3 Output | Response |
| --- | --- | --- | --- | --- |
| 1 | breast cancer risk model using logistic regression in United States | Breast Cancer / Logistic Regression / – / United States / Model Development | Breast Cancer / Logistic Regression / – / United States / Model Development | √ |
| 2 | lung cancer prediction with gene mutations and smoking, logistic, Chinacohort | Lung Cancer / Logistic Regression / Gene Mutation, Smoking Status / China / Model Development | Lung Cancer / Logistic Regression / Gene Mutation, Smoking Status / China / Model Development | √ |
| 3 | prostate cancer risk score based on PSA level in Germany | Prostate Cancer / Logistic Regression / PSA Level / Germany / Model Development | Prostate Cancer / Logistic Regression / PSA Level / Germany / Model Development | √ |
| 4 | colorectal cancer Cox model validation study in United Kingdom | Colorectal Cancer / Cox Proportional Hazards Regression / – / United Kingdom / Model Validation | Colorectal Cancer / Cox Proportional Hazards Regression / – / United Kingdom / Model Validation | √ |
| 5 | nomogram for elderly patients predicting cancer occurrence | – / Nomogram / Age / – / Model Development | – / Nomogram / – / – / Model Development | Missed “age” |
| 6 | endometrial cancer logistic prediction with BMI and hormone therapy in Japan | Endometrial Cancer / Logistic Regression / BMI, Hormone Therapy / Japan / Model Development | Endometrial Cancer / Logistic Regression / BMI, Hormone Therapy / Japan / Model Development | √ |
| 7 | gastric cancer CNN model developed in China | Gastric Cancer / Convolutional Neural Network / – / China / Model Development | Gastric Cancer / Convolutional Neural Network / – / China / Model Development | √ |
| 8 | patients with diabetes, pancreatic cancer risk prediction using alcohol intake in Korea | Pancreatic Cancer / – / Diabetes History, Alcohol Intake / Korea / Model Development | Pancreatic Cancer / – / Diabetes History, Alcohol Intake / Korea / Model Development | √ |
| 9 | BRCA1/2 mutation carriers risk model for ovarian cancer in Canada | Ovarian Cancer / – / BRCA1/2 Mutation / Canada / Model Development | Ovarian Cancer / – / BRCA1/2 Mutation / Canada / Model Development | √ |
| 10 | colorectal cancer logitic model validated in United States cohort (typo: “logistic”) | Colorectal Cancer / Logistic Regression / – / United States / Model Validation | Colorectal Cancer / Logistic Regression / – / United States / Model Validation | √ |
| 11 | developing a breast cancer risk prediction model using logistic regression that incorporates age, BMI, reproductive history, and family history in Australia | Breast Cancer / Logistic Regression / Age, BMI, Reproductive History, Family History / Australia / Model Development | Breast Cancer / Logistic Regression / Age, BMI, Reproductive History, Family History / Australia / Model Development | √ |
| 12 | lung cancer risk model combining smoking status, pack-years, genetic susceptibility, and environmental exposure using logistic regression in China | Lung Cancer / Logistic Regression / Smoking Status, Genetic Factors, Environmental Exposure / China / Model Development | Lung Cancer / Logistic Regression / Smoking Status, Genetic Factors, Environmental Exposure / China / Model Development | √ |
| 13 | prostate cancer prediction model based on PSA, age, and prior biopsy results using logistic regression | Prostate Cancer / Logistic Regression / PSA Level, Age, Prior Biopsy / – / Model Development | Prostate Cancer / Logistic Regression / PSA Level, Age, Prior Biopsy / – / Model Development | √ |
| 14 | validating a colorectal cancer Cox survival model that includes age, sex, BMI, and lifestyle factors | Colorectal Cancer / Cox Regression / Age, Sex, BMI, Lifestyle Factors / – / Model Validation | Colorectal Cancer / Cox Regression / Age, Sex, BMI, Lifestyle Factors / – / Model Validation | √ |
| 15 | ovarien cancer survival prediction using Cox regression with BRCA mutation status and clinical stage in France (typo: “ovarian”) | Ovarian Cancer / Cox Regression / BRCA Mutation, Clinical Stage / France / Model Development | Ovarian Cancer / Cox Regression / BRCA Mutation, Clinical Stage / France / Model Development | √ |
| 16 | endometrial cancer risk model using logistic regression integrating BMI, menopausal status, parity, and hormone replacement therapy | Endometrial Cancer / Logistic Regression / BMI, Menopausal Status, Parity, Hormone Therapy / – / Model Development | Endometrial Cancer / Logistic Regression / BMI, Menopausal Status, Parity, Hormone Therapy / – / Model Development | √ |
| 17 | CNN-based gastric cancer prediction model developed in China using imaging and clinical variables | Gastric Cancer / Convolutional Neural Network / Imaging Features, Clinical Factors / China / Model Development | Gastric Cancer / Convolutional Neural Network / Imaging Features, Clinical Factors / China / Model Development | √ |
| 18 | pancreatic cancer risk prediction model incorporating diabetes history, alcohol intake, smoking status, and age in Italy | Pancreatic Cancer / – / Diabetes History, Alcohol Intake, Smoking Status, Age / Italy / Model Development | Pancreatic Cancer / – / Diabetes History, Alcohol Intake, Smoking Status, Age / Italy / Model Development | √ |
| 19 | ovarian cancer risk assessment model for BRCA1/2 mutation carriers considering age and family history | Ovarian Cancer / – / BRCA1/2 Mutation, Age, Family History / – / Model Development | Ovarian Cancer / – / BRCA1/2 Mutation, Age, Family History / – / Model Development | √ |
| 20 | colorectal cancer logistic regression model evaluated in a United States population including diett, BMI, and family history (typo: “diet”) | Colorectal Cancer / Logistic Regression / Diet, BMI, Family History / United States / Model Validation | Colorectal Cancer / Logistic Regression / Diet, BMI, Family History / United States / Model Validation | √ |
| 21 | breast cancer Cox survival model incorporating age, tumor characteristics, and genetic risk score in Sweden | Breast Cancer / Cox Regression / Age, Tumor Features, Genetic Risk Score / Sweden / Model Development | Breast Cancer / Cox Regression / Age, Tumor Features, Genetic Risk Score / Sweden / Model Development | √ |
| 22 | lung cancer nomogram integrating smoking intensity, emphysema, and nodule characteristics | Lung Cancer / Nomogram / Smoking Intensity, Emphysema, Nodule Features / – / Model Development | Lung Cancer / Nomogram / Smoking Intensity, Emphysema, Nodule Features / – / Model Development | √ |
| 23 | random forest colorectal cancer risk model using dietary patterns, physical activity, and genetic markers in Netherlands | Colorectal Cancer / Random Forest / Diet, Physical Activity, Genetic Factors / Netherlands / Model Development | Colorectal Cancer / Random Forest / Diet, Physical Activity, Genetic Factors / Netherlands / Model Development | √ |
| 24 | neural network based prostate cancer prediction combining PSA, MRI features, and clinical indicators | Prostate Cancer / Neural Network / PSA Level, Imaging Features, Clinical Factors / – / Model Development | Prostate Cancer / Neural Network / PSA Level, Imaging Features, Clinical Factors / – / Model Development | √ |
| 25 | gastric cancer nomalgram including age, Helicobacter pylori infection, and smoking status in Japan (typo: “nomogram”) | Gastric Cancer / Nomogram / Age, H. pylori Infection, Smoking Status / Japan / Model Development | Gastric Cancer / Nomogram / Age, H. pylori Infection, Smoking Status / Japan / Model Development | √ |
| 26 | hepatocellular carcinoma survival model using Cox regression with AFP, cirrhosis, and viral hepatitis status in China | Hepatocellular Carcinoma / Cox Regression / AFP Level, Cirrhosis, Viral Hepatitis / China / Model Development | Hepatocellular Carcinoma / Cox Regression / AFP Level, Cirrhosis, Viral Hepatitis / China / Model Development | √ |
| 27 | bladder cancer risk prediction model integrating age, smoking history, occupational exposure, and sex | Bladder Cancer / – / Age, Smoking Status, Occupational Exposure, Sex / – / Model Development | Bladder Cancer / – / Age, Smoking Status, Occupational Exposure, Sex / – / Model Development | √ |
| 28 | thyroid cancer random forest model using ultrasound features, age, and gender | Thyroid Cancer / Random Forest / Imaging Features, Age, Sex / – / Model Development | Thyroid Cancer / Random Forest / Imaging Features, Age, Sex / – / Model Development | √ |
| 29 | head and neck cancer logistic model combining alcohol intake, smoking, and HPV status | Head & Neck Cancer / Logistic Regression / Alcohol Intake, Smoking Status, HPV Status / – / Model Development | Head & Neck Cancer / Logistic Regression / Alcohol Intake, Smoking Status, HPV Status / – / Model Development | √ |
| 30 | lymphoma Cox survival model incorporating age, stage, and treatment-related variables in Spain | Lymphoma / Cox Regression / Age, Disease Stage, Treatment Factors / Spain / Model Development | Lymphoma / Cox Regression / Age, Disease Stage, Treatment Factors / Spain / Model Development | √ |
| 31 | esophageal cancer risk prediction using logistic regression including age, smoking, alcohol consumption, and BMI | Esophageal Cancer / Logistic Regression / Age, Smoking Status, Alcohol Intake, BMI / – / Model Development | Esophageal Cancer / Logistic Regression / Age, Smoking Status, Alcohol Intake, BMI / – / Model Development | √ |
| 32 | melanoma risk model incorporating UV exposure, skin type, age, and family history | Melanoma / Cox Regression Model / UV Exposure, Skin Type, Age, Family History / – / Model Development | Melanoma / Cox Regression Model / UV Exposure, Skin Type, Age, Family History / – / Model Development | √ |
| 33 | pancreatic cancer Cox survival model including tumor stage, CA19-9 level, age, and treatment modality | Pancreatic Cancer / Cox Regression / Tumor Stage, CA19-9, Age, Treatment Factors / – / Model Development | Pancreatic Cancer / Cox Regression / Tumor Stage, CA19-9, Age, Treatment Factors / – / Model Development | √ |
| 34 | cervical cancer risk prediction model using logistic regression with HPV infection, age, parity, and smoking | Cervical Cancer / Logistic Regression / HPV Status, Age, Parity, Smoking Status / – / Model Development | Cervical Cancer / Logistic Regression / HPV Status, Age, Parity, Smoking Status / – / Model Development | √ |
| 35 | glioma survival prediction using Cox regression integrating age, tumor grade, and molecular markers | Glioma / Cox Regression / Age, Tumor Grade, Molecular Markers / – / Model Development | Glioma / Cox Regression / Age, Tumor Grade, Molecular Markers / – / Model Development | √ |
| 36 | liver cancer risk model for non-HCC patients using random forest with metabolic and clinical indicators | Liver Cancer (non-HCC) / Random Forest / Metabolic Factors, Clinical Indicators / – / Model Development | Liver Cancer (non-HCC) / Random Forest / Metabolic Factors, Clinical Indicators / – / Model Development | √ |
| 37 | prostate cancer nomogram incorporating PSA, age, prostate volume, and family history | Prostate Cancer / Nomogram / PSA Level, Age, Prostate Volume, Family History / – / Model Development | Prostate Cancer / Nomogram / PSA Level, Age, Prostate Volume, Family History / – / Model Development | √ |
| 38 | gastic and colon model using CNN (typo: “gastric”; missing keyword “cancer”) | Gastric Cancer, Colon Cancer / Convolutional Neural Network / – / – / Model Development | – / Convolutional Neural Network / – / – / Model Development | Missed cancer type |
| 39 | ovarian cancer risk prediction integrating CA125 level, age, menopausal status, and family history | Ovarian Cancer / – / CA125 Level, Age, Menopausal Status, Family History / – / Model Development | Ovarian Cancer / – / CA125 Level, Age, Menopausal Status, Family History / – / Model Development | √ |
| 40 | bladder cancer Cox survival model including stage, grade, smoking history, and treatment strategy | Bladder Cancer / Cox Regression / Tumor Stage, Tumor Grade, Smoking Status, Treatment Factors / – / Model Development | Bladder Cancer / Cox Regression / Tumor Stage, Tumor Grade, Smoking Status, Treatment Factors / – / Model Development | √ |

Supplement information 1.7 The evaluation results of the tag extraction tasks for DeepSeek-V3.

| Category | N (Gold) | TP | FP | FN | Precision | Recall | F1 | Wilson 95% CI (Precision) |
| --- | --- | --- | --- | --- | --- | --- | --- | --- |
| **Cancer Type** | 39 | 38 | 0 | 1 | 1.000 | 0.974 | 0.987 | [0.95, 1.00] |
| **Model Type** | 36 | 36 | 0 | 0 | 1.000 | 1.000 | 1.000 | [0.95, 1.00] |
| **Predictive Factors** | 35 | 34 | 0 | 1 | 1.000 | 0.971 | 0.985 | [0.95, 1.00] |
| **Country** | 20 | 20 | 0 | 0 | 1.000 | 1.000 | 1.000 | [0.91, 1.00] |
| **Research Type** | 40 | 40 | 0 | 0 | 1.000 | 1.000 | 1.000 | [0.95, 1.00] |
| **Micro Average** | 170 | 168 | 0 | 2 | 1.000 | 0.988 | 0.994 | – |
| **Macro Average** | – | – | – | – | 1.000 | 0.989 | 0.994 | – |

Supplemental Information 1.8 Prompts and query terms for different applications

|  | **CanRisk-RAG** | **ChatGPT-4o** | **Scholar AI** | **Gemini 1.5 Flash** | **PubMed** |
| --- | --- | --- | --- | --- | --- |
| **Theme 1** | Please provide me with cancer risk prediction models for [cancer type]. | Can you recommend 20 articles related to cancer risk prediction models for [cancer type]? Please provide information on the predictive factors, performance outcomes, modeling methods used, and metadata of the models, such as the article links, journal names and impact factors, publication years, and countries. | Can you recommend 20 articles related to cancer risk prediction models for [cancer type]? Please provide information on the predictive factors, performance outcomes, modeling methods used, and metadata of the models, such as the article links, journal names and impact factors, publication years, and countries. | Can you recommend 20 articles related to cancer risk prediction models for [cancer type]? Please provide information on the predictive factors, performance outcomes, modeling methods used, and metadata of the models, such as the article links, journal names and impact factors, publication years, and countries. | [cancer type] risk prediction models |
| **Theme 2** | Please provide me with cancer risk prediction models using [model type]. | Can you recommend 20 articles related to cancer risk prediction models using [model type]? Please provide information on the cancer types, predictive factors, performance outcomes, and metadata of the models, such as the article links, journal names and impact factors, publication years, and countries. | Can you recommend 20 articles related to cancer risk prediction models using [model type]? Please provide information on the cancer types, predictive factors, performance outcomes, and metadata of the models, such as the article links, journal names and impact factors, publication years, and countries. | Can you recommend 20 articles related to cancer risk prediction models using [model type]? Please provide information on the cancer types, predictive factors, performance outcomes, and metadata of the models, such as the article links, journal names and impact factors, publication years, and countries. | cancer risk prediction models AND [model type] |
| **Theme 3** | Please provide me with cancer risk prediction models with specific predictive factors, such as [predictive factors]. | Can you recommend 20 articles related to cancer risk prediction models with specific predictive factors, such as [predictive factors]? Please provide information on the cancer types, construction methods used, all predictive factors, performance outcomes, and metadata of the models, such as the article links, journal names and impact factors, publication years, and countries. | Can you recommend 20 articles related to cancer risk prediction models with specific predictive factors, such as [predictive factors]? Please provide information on the cancer types, construction methods used, all predictive factors, performance outcomes, and metadata of the models, such as the article links, journal names and impact factors, publication years, and countries. | Can you recommend 20 articles related to cancer risk prediction models with specific predictive factors, such as [predictive factors]? Please provide information on the cancer types, construction methods used, all predictive factors, performance outcomes, and metadata of the models, such as the article links, journal names and impact factors, publication years, and countries. | cancer risk prediction models AND [predictive factors] |
| **Theme 4** | Please provide me with cancer risk prediction models using [model type] for [cancer type] that include predictive factors, such as[predictive factors]. | Can you recommend 20 articles related to cancer risk prediction models using [model type] for [cancer type] that include predictive factors, such as[predictive factors]? Please provide information on the cancer types, construction methods used, all predictive factors, performance outcomes, and metadata of the models, such as the article links, journal names and impact factors, publication years, and countries. | Can you recommend 20 articles related to cancer risk prediction models using [model type] for [cancer type] that include predictive factors, such as[predictive factors]? Please provide information on the cancer types, construction methods used, all predictive factors, performance outcomes, and metadata of the models, such as the article links, journal names and impact factors, publication years, and countries. | Can you recommend 20 articles related to cancer risk prediction models using [model type] for [cancer type] that include predictive factors, such as[predictive factors]? Please provide information on the cancer types, construction methods used, all predictive factors, performance outcomes, and metadata of the models, such as the article links, journal names and impact factors, publication years, and countries. | [cancer type] risk prediction models AND [model type] AND [predictive factors] |

**Regarding the number of returned results, CanRisk-RAG allows configuration through a Top-K parameter. For PubMed, the "Best Match" mode returns the top 20 results by default. The other three large language model applications impose result limits based on the number of outputs requested within the input prompt.*

Supplemental Information 1.8 The scoring criteria for relevance, reliability, authenticity, data completeness, consistency.

| Score | Topic | Relevance | Reliability | Authenticity | Data Completeness | Consistency |
| --- | --- | --- | --- | --- | --- | --- |
| 10 | Theme1 | All retrieved literature must be related to cancer risk prediction models, and the cancer types involved must be highly relevant to the conditions of interest. | All literature is published in authoritative journals, and some models in the literature have high evaluation performance. | All retrieved literature is authentic and can be retrieved by various journal databases. | All retrieved literature contains complete model data, including metadata, cancer types, model construction types, model performance, predictors, etc. | With consistent search conditions, the model results obtained from two prompts or searches are entirely consistent. |
|  | Theme2 | All retrieved literature must be related to cancer risk prediction models, and the types of model construction involved must be highly relevant to the conditions of interest. |  |  |  |  |
|  | Theme3 | All retrieved literature must be related to cancer risk prediction models, and the predictor information involved must be highly relevant to the conditions of interest. |  |  |  |  |
|  | Theme4 | All retrieved literature must be related to cancer risk prediction models, and the information on cancer types, model construction types, and predictors involved must be highly relevant to the conditions of interest. |  |  |  |  |
| 9 | Theme1 | 90% of the retrieved literature must be related to cancer risk prediction models, and the cancer types involved must be highly relevant to the conditions of interest. | More than 15 papers are published in authoritative journals, and the models have high evaluation performance. | 90% of the literature is authentic and can be retrieved by various journal databases. | More than 50% of retrieved papers contain complete model data, including metadata, cancer types, model construction types, model performance, predictors, etc. | With consistent search conditions, the model results obtained from two prompts or searches are 90% consistent. |
|  | Theme2 | 90% of the retrieved literature must be related to cancer risk prediction models, and the types of model construction involved must be highly relevant to the conditions of interest. |  |  |  |  |
|  | Theme3 | 90% of the retrieved literature must be related to cancer risk prediction models, and the predictor information involved must be highly relevant to the conditions of interest. |  |  |  |  |
|  | Theme4 | More than 15 papers must be related to cancer risk prediction models, and the information on cancer types, model construction types, and predictors involved must be highly relevant to the conditions of interest. |  |  |  |  |
| 8 | Theme1 | 80% of the retrieved literature must be related to cancer risk prediction models, and the cancer types involved must be highly relevant to the conditions of interest. | More than 50% of the retrieved literature is published in authoritative journals with high evaluation performance. | 80% of the literature is authentic and can be retrieved by various journal databases. | Fewer than 50% of retrieved papers contain complete model data, including metadata, cancer types, model construction types, model performance, predictors, etc. | With consistent search conditions, the model results obtained from two prompts or searches are 80% consistent. |
|  | Theme2 | 80% of the retrieved literature must be related to cancer risk prediction models, and the types of model construction involved must be highly relevant to the conditions of interest. |  |  |  |  |
|  | Theme3 | 80% of the retrieved literature must be related to cancer risk prediction models, and the predictor information involved must be highly relevant to the conditions of interest. |  |  |  |  |
|  | Theme4 | More than 10 papers must be related to cancer risk prediction models, and the information on cancer types, model construction types, and predictors involved must be highly relevant to the conditions of interest. |  |  |  |  |
| 7 | Theme1 | 70% of the retrieved literature must be related to cancer risk prediction models, and the cancer types involved must be highly relevant to the conditions of interest. | Less than 50% of the retrieved literature is published in authoritative journals with high evaluation performance. | 70% of the literature is authentic and can be retrieved by various journal databases. | More than 50% of retrieved papers contain most of the model data, including metadata, cancer types, model construction types, model performance, predictors, etc. | With consistent search conditions, the model results obtained from two prompts or searches are 70% consistent. |
|  | Theme2 | 70% of the retrieved literature must be related to cancer risk prediction models, and the types of model construction involved must be highly relevant to the conditions of interest. |  |  |  |  |
|  | Theme3 | 70% of the retrieved literature must be related to cancer risk prediction models, and the predictor information involved must be highly relevant to the conditions of interest. |  |  |  |  |
|  | Theme4 | Fewer than 10 retrieved papers must be related to cancer risk prediction models, and the information on cancer types, model construction types, and predictors involved must be highly relevant to the conditions of interest. |  |  |  |  |
| 6 | Theme1 | 60% of the retrieved literature must be related to cancer risk prediction models, and the cancer types involved must be highly relevant to the conditions of interest. | More than 50% of the literature is published in authoritative journals. | 60% of the literature is authentic and can be retrieved by various journal databases. | Fewer than 50% of retrieved papers contain most of the model data, including metadata, cancer types, model construction types, model performance, predictors, etc. | With consistent search conditions, the model results obtained from two prompts or searches are 60% consistent. |
|  | Theme2 | 60% of the retrieved literature must be related to cancer risk prediction models, and the types of model construction involved must be highly relevant to the conditions of interest. |  |  |  |  |
|  | Theme3 | 60% of the retrieved literature must be related to cancer risk prediction models, and the predictor information involved must be highly relevant to the conditions of interest. |  |  |  |  |
|  | Theme4 | More than 10 retrieved papers must be related to cancer risk prediction models, and the information on cancer types, model construction types, and two types of predictors involved must be relevant to the conditions of interest. |  |  |  |  |
| 5 | Theme1 | 50% of the retrieved literature must be related to cancer risk prediction models, and the cancer types involved must be highly relevant to the conditions of interest. | 50% of the literature is published in authoritative journals. | 50% of the literature is authentic and can be retrieved by various journal databases. | More than 50% of retrieved papers contain some of the model data, including metadata, cancer types, model construction types, model performance, predictors, etc. | With consistent search conditions, the model results obtained from two prompts or searches are 50% consistent. |
|  | Theme2 | 50% of the retrieved literature must be related to cancer risk prediction models, and the types of model construction involved must be highly relevant to the conditions of interest. |  |  |  |  |
|  | Theme3 | 50% of the retrieved literature must be related to cancer risk prediction models, and the predictor information involved must be highly relevant to the conditions of interest. |  |  |  |  |
|  | Theme4 | 5-10 retrieved papers must be related to cancer risk prediction models, and the information on cancer types, model construction types, and two types of predictors involved must be relevant to the conditions of interest. |  |  |  |  |
| 4 | Theme1 | 40% of the retrieved literature must be related to cancer risk prediction models, and the cancer types involved must be highly relevant to the conditions of interest. | 40% of the retrieved literature is published in authoritative journals. | 40% of the literature is authentic and can be retrieved by various journal databases. | Fewer than 50% of retrieved papers contain some of the model data, including metadata, cancer types, model construction types, model performance, predictors, etc. | With consistent search conditions, the model results obtained from two prompts or searches are 40% consistent. |
|  | Theme2 | 40% of the retrieved literature must be related to cancer risk prediction models, and the types of model construction involved must be highly relevant to the conditions of interest. |  |  |  |  |
|  | Theme3 | 40% of the retrieved literature must be related to cancer risk prediction models, and the predictor information involved must be highly relevant to the conditions of interest. |  |  |  |  |
|  | Theme4 | 4 retrieved papers must be related to cancer risk prediction models, and the information on cancer types, model construction types, and two types of predictors involved must be relevant to the conditions of interest. |  |  |  |  |
| 3 | Theme1 | 30% of the retrieved literature must be related to cancer risk prediction models, and the cancer types involved must be highly relevant to the conditions of interest. | 30% of the literature is published in authoritative journals. | 30% of the literature is authentic and can be retrieved by various journal databases. | More than 50% of retrieved papers contain only basic metadata, including DOI numbers, literature links, publication years, titles, etc. | With consistent search conditions, the model results obtained from two prompts or searches are 30% consistent. |
|  | Theme2 | 30% of the retrieved literature must be related to cancer risk prediction models, and the types of model construction involved must be highly relevant to the conditions of interest. |  |  |  |  |
|  | Theme3 | 30% of the retrieved literature must be related to cancer risk prediction models, and the predictor information involved must be highly relevant to the conditions of interest. |  |  |  |  |
|  | Theme4 | 3 retrieved papers must be related to cancer risk prediction models, and the information on cancer types, model construction types, and two types of predictors involved must be relevant to the conditions of interest. |  |  |  |  |
| 2 | Theme1 | 20% of the retrieved literature must be related to cancer risk prediction models, and the cancer types involved must be highly relevant to the conditions of interest. | 20% of the literature is published in authoritative journals. | 20% of the literature is authentic and can be retrieved by various journal databases. | Fewer than 50% of retrieved papers contain only basic metadata, including DOI numbers, literature links, publication years, titles, etc. | With consistent search conditions, the model results obtained from two prompts or searches are 20% consistent. |
|  | Theme2 | 20% of the retrieved literature must be related to cancer risk prediction models, and the types of model construction involved must be highly relevant to the conditions of interest. |  |  |  |  |
|  | Theme3 | 20% of the retrieved literature must be related to cancer risk prediction models, and the predictor information involved must be highly relevant to the conditions of interest. |  |  |  |  |
|  | Theme4 | 2 retrieved papers must be related to cancer risk prediction models, and the information on cancer types, model construction types, and two types of predictors involved must be relevant to the conditions of interest. |  |  |  |  |
| 1 | Theme1 | 10% of the retrieved literature must be related to cancer risk prediction models, and the cancer types involved must be highly relevant to the conditions of interest. | 10% of the literature is published in authoritative journals. | 10% of the literature is authentic and can be retrieved by various journal databases. | Fewer than 25% of retrieved papers contain only basic metadata, including DOI numbers, literature links, publication years, titles, etc. | With consistent search conditions, the model results obtained from two prompts or searches are 10% consistent. |
|  | Theme2 | 10% of the retrieved literature must be related to cancer risk prediction models, and the types of model construction involved must be highly relevant to the conditions of interest. |  |  |  |  |
|  | Theme3 | 10% of the retrieved literature must be related to cancer risk prediction models, and the predictor information involved must be highly relevant to the conditions of interest. |  |  |  |  |
|  | Theme4 | 1 retrieved paper must be related to cancer risk prediction models, and the information on cancer types, model construction types, and two types of predictors involved must be relevant to the conditions of interest. |  |  |  |  |
| 0 | Theme1 | All retrieved literature is unrelated to cancer risk prediction models, or the cancer types involved are irrelevant to the conditions of interest. | All retrieved literature is not published in authoritative journals. | All retrieved literature lacks authenticity and cannot be retrieved by various journal databases. | All retrieved literature lacks any relevant metadata and model data. | With consistent search conditions, the model results obtained from two prompts or searches are completely inconsistent. |
|  | Theme2 | All retrieved literature is unrelated to cancer risk prediction models, or the types of model construction involved are irrelevant to the conditions of interest. |  |  |  |  |
|  | Theme3 | All retrieved literature is unrelated to cancer risk prediction models, or the predictor information involved is irrelevant to the conditions of interest. |  |  |  |  |
|  | Theme4 | All retrieved literature is unrelated to cancer risk prediction models, or only one type of information among cancer types, model construction types, or predictors is relevant to the conditions of interest, or none are relevant at all. |  |  |  |  |

Supplement information 1.10 The validation dataset of 20 queries for model recommendation benchmarking.

| ***Themes*** | **Subthemes** |
| --- | --- |
| *Model Recommendation tasks Based on Cancer Type* | Colorectal Cancer |
|  | Ovarian Cancer |
|  | Gastric Cancer |
|  | Pancreatic Cancer |
|  | Hepatocellular Carcinoma |
| *Model Recommendation tasks Based on Model Type* | Cox Proportional Hazards Regression |
|  | Nomogram |
|  | Decision Tree |
|  | Random Forest |
|  | Risk Score |
| *Model Recommendation tasks Based on Predictive factors* | BMI, Alcohol Intake, Lifestyle Factors |
|  | Genetic Susceptibility, Clinical Indicators |
|  | Environmental Exposure, Smoking Status |
|  | Biochemical Markers, Liver Function Indicators |
|  | Reproductive History, Hormonal Factors |
| *Comprehensive Model Recommendation tasks* | Cancer Type: Breast Cancer; Modeling type: Logistic Regression; Predictive factors: Age, Family History, BMI |
|  | Cancer Type: Lung Cancer; Modeling type: Neural Network; Predictive factors: Smoking Status, Gene Mutation, Age |
|  | Cancer Type: Prostate Cancer; Modeling type: Support Vector Machine; Predictive factors: PSA Level, Prior Biopsy |
|  | Cancer Type: Gastric Cancer; Modeling type: Cox Proportional Hazards Regression; Predictive factors: Smoking Status, Alcohol Intake, Helicobacter pylori Infection |
|  | Cancer Type: Hepatocellular Carcinoma; Modeling type: Random Forest; Predictive factors: Biochemical Markers, Viral Infection History |

Supplemental Information 1.11 The scoring criteria for usability metrics.

| Metric | Scoring Criteria |
| --- | --- |
| User Goal Achievement | 3 - Excellent: The recommended models fully meet the user's personalized needs, with the recommendations being highly aligned with the specific query (e.g., specific cancer type, modeling type, included predictive factors). The recommended results comprehensively cover all key elements of interest to the user, requiring minimal additional filtering.  2 -Good: The recommended models partially meet the user's personalized needs, covering most of the key aspects of the query (e.g., specific cancer type, modeling type, included predictive factors), but there are some omissions or inaccuracies in certain areas. The user would need to perform a moderate level of filtering or supplementation to fully satisfy their requirements.  1 - Needs Improvement: The recommended models largely fail to meet the user's personalized needs. The recommendations deviate from the intended query, lacking critical attributes (e.g., failure to cover the specified cancer type, mismatched modeling types, or predictive factors). The user would need to conduct a new search or perform extensive filtering to identify models that meet their requirements. |
| Operative accessibility | 3 - Excellent: The website has a clear and attractive design with a well-organized layout that makes it easy for users to find and use the necessary features. The site loads very quickly. It performs well across various devices, including desktops and mobile devices.  2 - Good: The site has a fairly simple and clear design, but it may take some time for users to adapt or find specific features. Loading speeds are generally good, although some content-rich pages may load slightly slower. The website performs well on mobile devices, with most features functioning correctly, though some design elements may not be fully optimized for mobile use.  1 - Needs Improvement: The website's design may be overly complex or outdated. Features are not clearly indicated, requiring users to spend considerable time understanding how to use them. Loading speeds are slow, especially on content-heavy pages, which may affect the browsing experience. The site has poor compatibility with mobile devices, lacking responsive design, which leads to inconvenience when used on smaller screens. |

Supplemental Information 1.12 The answer examples of Scholar AI

Answer 1

Meads et al. (2012)
Title: A Systematic Review of Breast Cancer Incidence Risk Prediction Models
Focus: UK-based validation, modifiable risk factors
Journal: Breast Cancer Research and Treatment

Answer 2

A systematic review of breast cancer incidence risk prediction models with meta-analysis of their performance

Authors: C. Meads, I. Ahmed, R.D. Riley

Journal: Breast Cancer Research and Treatment

Year: 2012 | Country: UK

Focus: Meta-analysis of models like Gail, Tyrer-Cuzick, BRCAPRO; shows Tyrer-Cuzick offers superior discrimination.

Methods: Meta-analysis of AUCs

Factors: Lifestyle and genetic risk

**Key information such as modeling methods and predictive factors is either inaccurately captured or entirely omitted. Moreover, when repeated recommendation requests are made, inconsistencies in the content of the same article also arise.*

Supplemental Information 1.13 The answer examples of Gemini 1.5 Flash

Answer 1

-Article: A deep learning model to predict breast cancer risk from mammograms.

Journal: Journal of Clinical Oncology (Impact Factor: 50.7)

-Publication Year: 2019

-Country: USA

-Article Link: [https://ascopubs.org/doi/full/10.1200/JCO.19.00248](https://www.google.com/search?q=https://ascopubs.org/doi/full/10.1200/JCO.19.00248)

-Predictive Factors: Raw pixel data from digital mammograms. Some versions also incorporate traditional clinical risk factors.

-Performance Outcomes: Demonstrates superior performance to traditional models, with AUCs reported to be around 0.75 for predicting breast cancer within 5 years.

-Modeling Method: A deep learning model, specifically a convolutional neural network (CNN), trained on a large dataset of mammograms and patient outcomes.

**Article Link: https://ascopubs.org/doi/full/10.1200/JCO.19.00248 does not exist. Therefore, the other related metadata are also fabricated.*

Answer 2

-Title:Breast Cancer Risk Models May Incorrectly Classify Many Women

-Predictive Factors: The article focuses on three widely used models: the Breast Cancer Risk Assessment Tool (BCRAT, or the Gail model), the Breast Cancer Surveillance Consortium (BCSC), and the International Breast Intervention Study (IBIS, or the Tyrer-Cuzick model). These models typically use a combination of factors like age, personal and family history, reproductive history, and breast density.

-Modeling Method: It compares the performance of established statistical models, pointing out their limitations when applied to individuals rather than populations.

-Performance Outcomes: The study found that while the models had similar accuracy at the population level, they often disagreed on which individual women were at "high risk," especially at the ≥3.0% 5-year risk threshold recommended by the U.S. Preventive Services Task Force. This can lead to both false positives and missed cases.

-Source: UCLA Health Newsroom

-Publication Year: 2023

-Country: United States

-Link: <https://www.uclahealth.org/news/release/ElmoreBCRisk2023>

**Classified as news.*

Supplemental Information 1.14 CanRisk-RAG’s usability of score results

|  | User goal achievement | Operative accessibility |
| --- | --- | --- |
| User 1 | 3 | 3 |
| User 2 | 3 | 3 |
| User 3 | 3 | 2 |
| User 4 | 2 | 2 |
| User 5 | 3 | 2 |
| User 6 | 2 | 2 |
| User 7 | 2 | 3 |
| User 8 | 3 | 3 |
| User 9 | 3 | 2 |
| User 10 | 3 | 3 |
| Average score | 2.7 ± 0.48（Mean±SD） | 2.5±0.53（Mean SD） |
